# Integration of bulk and single-cell transcriptomic data reveals a novel signature related to liver metastasis and basement membrane in pancreatic cancer

**DOI:** 10.3389/fimmu.2025.1671956

**Published:** 2025-10-29

**Authors:** Dongkai Zhou, Cheng Zhong, Qifan Yang, Bijun Cui, Yizhi Wang

**Affiliations:** ^1^ Department of Hepatobiliary and Pancreatic Surgery, The Second Affiliated Hospital, Zhejiang University School of Medicine, Hangzhou, Zhejiang, China; ^2^ Key Laboratory of Precision Diagnosis and Treatment for Hepatobiliary and Pancreatic Tumor of Zhejiang Province, Hangzhou, Zhejiang, China

**Keywords:** pancreatic cancer, liver metastasis, basement membrane, prognostic model, immunotherapy response, ScRNA-seq

## Abstract

**Background:**

Pancreatic cancer (PC) is characterized by an exceptionally poor prognosis, primarily attributable to its aggressive metastatic behavior and high recurrence rates. Liver metastasis is the predominant distant metastasis model of PC. Moreover, invasion and metastasis of PC are closely associated with the remodeling or loss of basement membrane (BM). Consequently, identifying pivotal genes involved in PC liver metastasis (PCLM) and BM could pave the way for more effective and precise targeted therapies. This study aims to construct a prognostic model based on PCLM and BM-related genes, while also validating the association between this model and the immune microenvironment of PC, as well as its predictive value for the efficacy of chemotherapy and immunotherapy.

**Methods:**

Transcriptomic, mutation, and clinical data were retrieved from the TCGA, ICGC, and GEO databases. Core prognostic genes were identified through single-cell (sc) and bulk transcriptomic sequencing data combined with WGCNA analysis. The prognostic model was established using machine learning algorithms and multivariate Cox regression analyses. Specifically, the TCGA-PAAD cohort was utilized as the training set while the PACA-AU cohort served as the validation set. The performance of this model was assessed in both the training and validation sets. Additionally, the associations between the model and tumor mutation burden (TMB) as well as tumor immunity were evaluated using multiple immunity databases. Additionally, the predictive capacity of the model regarding the efficacy of chemotherapy, immunotherapy, and targeted therapy was also assessed. Finally, the expression of COL7A1 was knockdown in cancer-associated fibroblasts (CAFs) in PC to explore its role in PC progression.

**Results:**

30 PCLM and BM-related prognostic genes were preliminarily identified integrating sc and bulk transcriptomic sequencing data. Through machine learning algorithms and multivariate Cox regression analysis, six signatures, including COL7A1, ITGA6, ITGA7, ITGB5, ITGB7 and NTN4, were subsequently utilized to construct a prognostic model. This model demonstrated superior prognostic performance compared with conventional clinicopathological variables. Immune analysis revealed that the infiltration levels of M0 macrophages and Treg cells were significantly elevated in the high-risk group, whereas the infiltration levels of CD8+T cells and γδT cells were significantly reduced. Moreover, the high-risk group exhibited higher TMB and poorer survival outcomes. Additionally, the high-risk group showed a higher TIDE and a lower IPS score, indicating less effective immunotherapy response. Furthermore, the high-risk group displayed significantly higher IC50 values for common PC chemotherapeutics, suggesting reduced chemotherapeutic efficacy. Notably, scRNA-seq analysis indicated that COL7A1, which has not been systematically investigated in PC previously, predominantly expressed in fibroblasts. Specifically, CAFs exhibited significantly higher expression levels of COL7A1 compared to normal pancreatic fibroblasts, and COL7A1 knockdown in CAFs markedly reduced the migratory capacity of PC cells while enhancing their chemosensitivity to gemcitabine.

**Conclusion:**

This study developed and rigorously validated an innovative prognostic model for PC. This model, incorporating pivotal genes of PCLM and BM, may also serve as potential tool for predicting the tumor immune microenvironment and therapeutic efficacy. Notably, COL7A1, which was demonstrated to be vital in PC metastasis in this study, warrants further investigation in future research.

## Introduction

Pancreatic cancer (PC) is currently the fourth leading cause of cancer-related deaths in the US and is projected to become the second leading cause by 2030 (1, 2). Despite decades of efforts, the 5-year overall survival (OS) rate has only increased from 4% to 13%, with more than half of patients presenting metastatic disease at diagnosis, resulting in dismal prognosis (1). Liver metastasis is a common mode of dissemination for PC and is associated with an even worse outcome (3). The underlying mechanisms of pancreatic cancer liver metastasis (PCLM) are complicated, with the interaction between tumor cells and the tumor microenvironment (TME) being a vital factor contributing to PCLM and treatment resistance (4). Gemcitabine plus nab-paclitaxel and FOLFIRINOX remain the standard first-line therapies for metastatic PC (5, 6). However, conventional chemotherapies have limited efficacy in PC, particularly in PCLM, with only 1% of patients surviving beyond 5 years (7). Immunotherapy, while highly effective in treating various cancers, has shown limited success in PC. Pembrolizumab demonstrates efficacy only in a small subset of patients with microsatellite instability-high (MSI-H) PC (8). A phase II trial combining dual immune checkpoint inhibitors with gemcitabine plus nab-paclitaxel also yielded unsatisfactory results (9).The immunosuppressive nature of the TME and low tumor mutation burden may contribute to the failure of immunotherapy in PC (10). Additionally, targeted therapy based on specific biomarkers can only benefit certain subgroups of PC patients (11). Therefore, developing models to identify patients who are most likely to benefit from these therapies will enhance precision medicine approaches for PC and reduce unnecessary medical costs.

The basement membrane (BM) is a thin, dense layer of extracellular matrix (ECM) that plays a critical role in normal tissue development and function (12). The two primary components of BM, laminin and collagen IV, are responsible for transmitting cellular signals and maintaining structural integrity. Epithelial tumor cells must invade BM to achieve blood and lymphatic metastasis, which accounts for the majority of cancer-related deaths (13). Mechanistically, protease-dependent BM degradation, such as matrix metalloproteinases (MMPs), cysteine proteases, and serine proteases, not only weakens its barrier function but also enhances cell migration through signaling pathways activated by cleavage products (14). Additionally, heterotypic cell interactions, including those involving immune cells, fibroblasts, and myoepithelial cells, can degrade or remodel the BM, thereby promoting tumor cell metastasis via various signaling pathways (15–17). Previous studies have highlighted the role of BM-related genes in the progression and prognosis of PC. Lin et al. constructed and validated a seven-gene BM-related model that accurately predicts outcomes in PC patients and explored the malignant behavior of TINAG expression (18). Subsequently, Zhang et al. identified DSG3, MET, and PLAU to construct a PC prognostic model that not only predicted patient survival but also correlated with specific immune cell infiltration. They further validated the efficacy of their model using an external cohort (19). Despite these advances, there remains a lack of research combining liver metastasis and BM-related genes using bulk and single-cell transcriptomic data to develop a comprehensive and effective prognostic model for PC.

Immune cell infiltration in TME has been extensively reported to play a critical role in PC metastasis and immunotherapy outcomes. Tumor-associated neutrophils (TANs) can facilitate PCLM via multiple mechanisms, including angiogenesis, immune suppression and escape, as well as epithelial-mesenchymal transition (EMT) (20). Additionally, the M2 phenotype of tumor-associated macrophages (TAMs) enhances tumor cell migration, invasion, and self-renewal. However, disrupting the gal-9/dectin-1 interaction on the surface of M2 macrophages can reduce regulatory T cell (Treg) infiltration and reverse the immunosuppressive tumor microenvironment, thereby inhibiting tumor growth (21). PC is often considered an immunologically ‘cold’ tumor due to its poor response to immunotherapy (22). The ineffectiveness of immune checkpoint blockade (ICB) in PC is attributed to the low proportion of tumor-infiltrating T cells and the low tumor mutation burden (TMB) in PCs (23, 24). Nevertheless, a small subset of PC patients may benefit significantly from ICB therapy due to their unique patterns of immune cell infiltration. Thus, developing a model to predict the TME status in PC patients could aid in personalized treatment strategies and improve therapeutic efficacy.

Previous studies have indicated the critical role of BM-related genes in predicting TME status and synthetic therapy responses. Zhang et al. demonstrated that a BM-related prognostic model, comprising DSG3, MET, and PLAU, was associated with immune cell infiltration and the efficacy of chemotherapy and immunotherapy (19). Additionally, The study of Zhou et al. developed another BM-related gene scoring system to predict the immune microenvironment and treatment outcomes (25). However, whether immune cell infiltration in the TME can modulate BM to enhance PCLM remains unclear. Therefore, it is essential to construct a model integrating metastasis and BM-related genes to improve personalized immunotherapies and enhance the prognosis of PC patients. In this study, we developed a prognostic model based on metastasis and BM-related genes that can effectively predict the prognosis, immune microenvironment, and therapeutic outcomes for PC patients. This model may assist clinicians in providing personalized treatment strategies.

## Methods and materials

### Data obtainment

RNA-Seq data from 182 patients with pancreatic adenocarcinoma (PAAD) were obtained from the TCGA database (https://portal.gdc.cancer.gov/), comprising 178 tumor samples and 4 matched normal pancreatic tissue samples. Transcriptomic data from normal pancreatic tissues of 167 individuals were retrieved from the GTEx database (https://www.gtexportal.org). Gene expression microarray datasets (GSE71729 and GSE34153) and single-cell RNA sequencing datasets (GSE154778 and GSE197177) were downloaded from the GEO repository (http://www.ncbi.nlm.nih.gov/geo). Gene expression profiles for the PACA_AU cohort were acquired from the ICGC data portal (http://xena.ucsc.edu). A total of 178 TCGA_PAAD patients with complete clinical and transcriptomic data were designated as the training cohort, while 255 patients from the PACA_AU cohort were used as the independent validation cohort. Batch effects between TCGA_PAAD and PACA_AU datasets were corrected using the sva R package. Additionally, 224 BM-related genes were extracted from a previous study (26). Detailed characteristics of all datasets included were summarized in **Supplementary Table S1**.

### Identification of liver metastasis and BM related genes

To ensure robust identification of candidate genes exhibiting consistent differential expression across single-cell, bulk, primary, and metastatic contexts, both bulk and single-cell RNA sequencing data from primary and metastatic pancreatic tissues were integrated to identify genes associated with PCLM. The analytical workflow was as follows: Initial quality control of scRNA-seq data was performed using the following thresholds: 1, 000 ≤ nCount_RNA ≤ 30, 000, 200 ≤ nFeature_RNA ≤ 10, 000, percent.mt ≤ 20%, and percent.rb ≤ 50%. Subsequently, a resolution parameter of 1.5 was applied in the RNA_snn algorithm for cell clustering. Cell cluster visualization was conducted using both t-SNE and UMAP dimensionality reduction techniques. Cluster annotation was performed using the SingleR R package (Version 2.6.0) and manual curation based on canonical marker gene expression, including CD3D, CD3E, TRAC (T cells), KLRD1, GNLY, NKG7 (NK cells), CD19, CD79A, MS4A1 (B cells), EPCAM, KRT18, KRT19 (epithelial cells), CD68, CD163 (macrophages/monocytes), COL1A1, COL3A1 (fibroblasts), VWF, PECAM1, PLPP1 (endothelial cells), KIT, TPSAB1, TPSB2 (mast cells). WGCNA (Version 1.72-5) was employed to identify co-expression modules associated with PCLM in the GSE71729 and GSE34153 datasets. The optimal β parameter was determined using the pickSoftThreshold function based on Pearson correlation coefficients. Subsequently, co-expression modules were constructed using the blockwiseModules function. Furthermore, the weighted adjacency matrix was transformed into a topological overlap matrix (TOM), and the corresponding dissimilarity matrix was calculated as (1-TOM). The dynamic tree cutting approach was employed to conduct the module identification. The TOM type was set to “unsigned, “ and a minimum module size of 30 was applied to ensure the identification of biologically meaningful functional modules. Differentially expressed genes (DEGs) between primary tumor cells and liver metastatic cells were identified using the Seurat package (Version 5.1.0) in scRNA-seq datasets. Additionally, DEGs between normal pancreatic tissues and PC tissues, as well as between primary and metastatic tumor tissues, were analyzed using the limma R package (Version 3.60.3) in bulk RNA-seq datasets.

### Construction and validation of a novel prognostic model

To develop a novel prognostic model of PC based on metastasis-associated BM genes, a combination of 10 machine learning algorithms (CoxBoost, Lasso, stepwise Cox, plsRcox, Ridge, Enet, survival support vector machine (SurvivalSVM), generalized boosted regression models (GBMs), supervised principal components (SuperPC) and random forest (RSF)) were employed to screen for prognostic genes using Mime1 R (Version 0.0.0.9000) package. The detailed parameters of the 10 machine learning algorithms are provided in the original code and **Supplementary Table S2**. Subsequently, the gene coefficients were calculated using multivariate Cox regression analysis. The risk score for each patient was determined using the following formula: Risk score=Σ (Coef×Exp). Thereafter, patients in the training cohort were stratified into high- and low-risk groups based on the median value of the risk score. The same formula and cutoff value were also applied to stratify patients in the validation cohort. Then, a nomogram including AJCC tumor-node-metastasis (TNM) staging, grade, gender, age and risk score was constructed. To evaluate the accuracy and consistency of the prognostic model, receiver operating characteristic (ROC) curves, calibration curves, and decision curve analysis (DCA) curves were analyzed using the timeROC (Version 0.4), ggDCA (Version 1.2), survival (Version 3.6-4), and rms R (Version 6.9-0) packages.

### Functional enrichment and gene set enrichment analyses

Gene Ontology (GO) and Kyoto Encyclopedia of Genes Genomes (KEGG) analyses were conducted to compare the high- and low-risk groups of patients with PC using the clusterProfiler (Version 4.12.0) and enrichplot (Version 1.24.0) R packages. The enriched pathways in both the high- and low-risk groups were also identified using gene set enrichment analyses (GSEA) using the aforementioned R packages and The top5 signaling pathways were presented for each group. Additionally, the correlations between risk scores, prognostic genes, and KEGG pathways in tumors were analyzed using Gene Set Variation Analysis (GSVA) with the GSVA (Version 2.1.3) R package.

### Tumor mutation burden and drug sensitivity analyses

The mutation information of patients was retrieved from TCGA database. The gene mutation status in high- and low-risks group were analyzed using maftools (Version 2.20.0) R package. The survival analysis between different risks and tumor mutation burden (TMB) were explored via suvminer (Version 0.4.9) R package. The drug sensitivity analysis between high- and low-risk groups was performed by oncoPredict (Version 1.2) R package.

### Immune microenvironment analysis and immunotherapy

The single-sample GSEA (ssGSEA) algorithm was used to quantify the abundance of 16 immune cell types in each PC sample through GSVA (Version 2.1.3) R package (27). The immune profiles of the high- and low-risk groups were further characterized by applying multiple algorithms, including xCell (28), Estimating the Proportion of Immune and Cancer cells (EPIC) (29), Cell-type Identification By Estimating Relative Subsets Of RNA Transcripts (CIBERSORT) (30), Quantifying Tumor Immune Signature Events (QUANTISEQ) (31), and Microenvironment Cell Populations (MCP) counter (32). Additionally, the differential expression of immune checkpoint genes between the two groups was assessed to evaluate treatment sensitivity. Furthermore, Tumor Immune Dysfunction and Exclusion (TIDE) data for the TCGA cohort in both high- and low-risk groups were analyzed to acquire TIDE scores, immune exclusion scores, dysfunction scores, IPS scores, and MDSC scores through the TIDE website (http://tide.dfci.harvard.edu/) and then visualized through ggplot2 (Version 3.5.2) R package (33, 34).

### Cell culture and clinical specimens

PANC-1 and BxPC-3 PC cell lines were obtained from the American Type Culture Collection (ATCC, Manassas, VA, USA). PANC-1 cells were cultured in Dulbecco’s Modified Eagle’s Medium (DMEM, Hyclone, Logan, UT, USA), whereas BxPC-3 cells were maintained in Roswell Park Memorial Institute (RPMI) 1640 medium (Hyclone, Logan, UT, USA) supplemented with 10% fetal bovine serum (FBS, Gibco, CA, USA) under standard culture conditions (37 °C, 5% CO_2_). In addition, PC tissues and their corresponding normal pacnreatic tissues were collected from patients who underwent surgical resection at the Second Affiliated Hospital of Zhejiang University (SAHZU) and were used for further analysis.

### Extraction of pancreatic cancer-associated fibroblasts

Surgically resected PC tissues and adjacent normal pancreatic tissues were collected and immediately immersed in phosphate-buffered saline (PBS). The tissues were then thoroughly washed five times in a 50 ml tube using 10–20 ml of PBS per wash. Subsequently, ophthalmic scissors were used to carefully remove adipose tissue and other non-target tissues surrounding the PC and normal pancreatic tissues. The specimens were cut into 1–2 mm³ fragments and transferred into a 0.1% type II collagenase solution.The digestion was carried out at 37 °C in a shaker incubator with 5% CO_2_ for 4 hours. Following digestion, the suspension was filtered through a 4 μm cell strainer to remove undigested tissue debris, and the filtrate was collected in a 15 mL centrifuge tube. The sample was centrifuged at 1000 rpm for 5 minutes, washed twice with PBS, and the supernatant was discarded. The isolated cancer-associated fibroblasts (CAFs) were cultured in 6-well plates containing DMEM supplemented with 20% FBS and a penicillin-streptomycin solution. The cells were used for cytological experiments after 3–6 passages.

### CAFs transfection

CAFs were cultured in 24-well plates and transfected with previously RNA interference lentiviral vectors (Genechem, China) or a negative control (empty plasmid) for 24h. The lentiviral interference sequences used were as follows: COL7A1-shRNA(134364-1): GGAAACTCCACTTGCTGTTCC;COL7A1-shRNA(134365-2):GCAGCTCATCTGTCACCATTA; COL7A1-shRNA(134366-1): GCATCCAGCTACATCCTATCC. Following transfection, the medium was replaced with complete culture medium, and cells were cultured for an additional week. Subsequently, the medium was changed to complete medium supplemented with puromycin. After 72 hours, fluorescence intensity was assessed under a fluorescence microscope, and visible cellular fluorescence indicated successful transfection. As the lentivirus conferred puromycin resistance, stable lentiviral-expressing cell lines were selected by puromycin supplementation in the culture medium. During this period, cells were gradually passaged from 24-well plates to 12-well plates and finally to 6-well plates due to confluence.

### Co-culture of PC cells and CAFs

**Figure 1:**
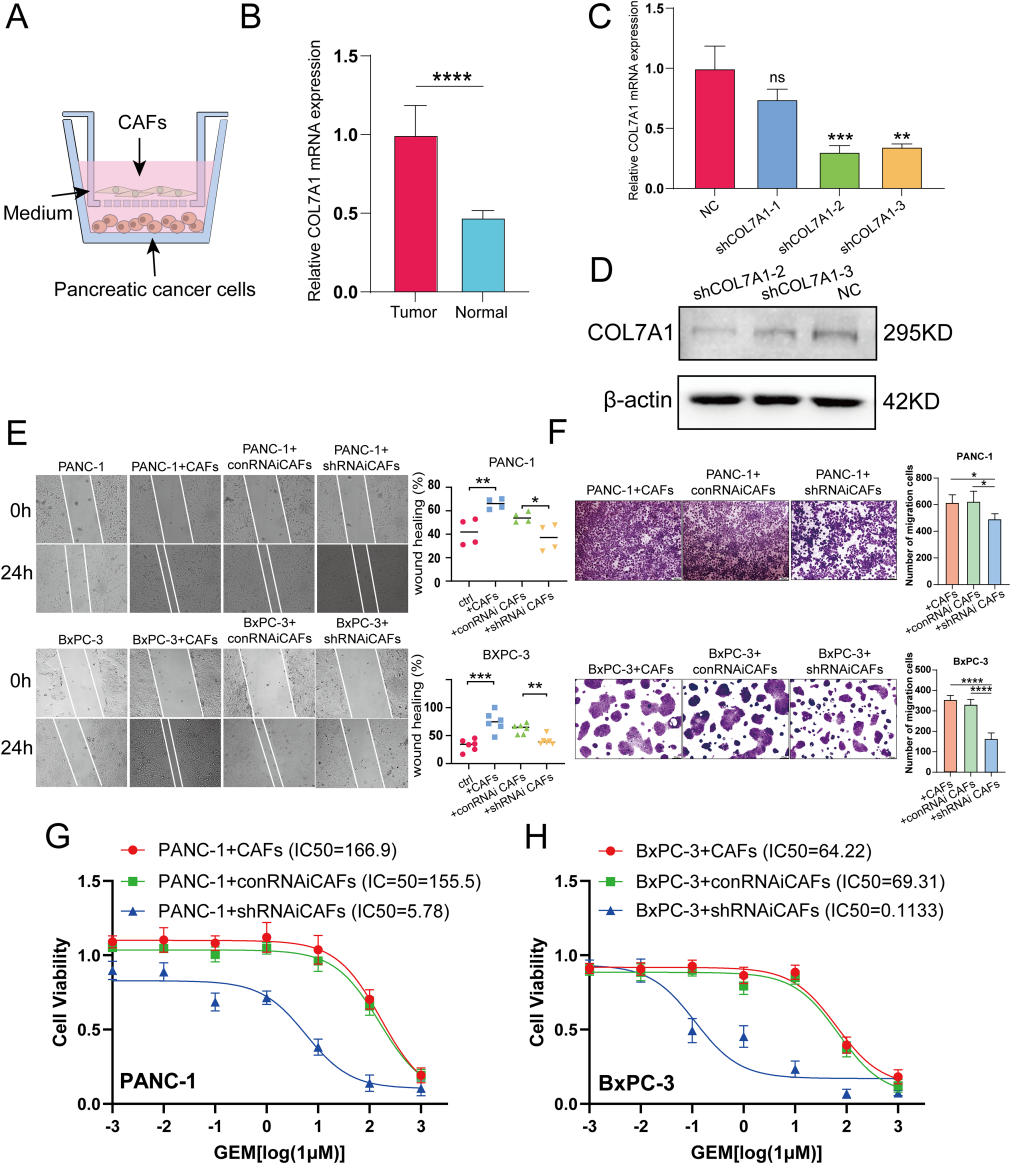
**(A)** Schematic diagram of co-culture of CAFs and PC cells. **(B)** The mRNA levels of COL7A1 between CAFs and normal pancreatic fibroblsts. **(C, D)** qRT-PCR and western blot analyses evaluation of COL7A1 lentiviral knockdown efficiency. **(E)** The results of wound healing assay using PANC-1 and BxPC-3 PC cells. **(F)** The results of transwell assay using PANC-1 and BxPC-3 PC cells. **(G)** IC50 curves under gradient concentration GEM after COL7A1 knockdown in CAFs co-cultured with in PANC-1 cells. **(H)** IC50 curves under gradient concentration GEM after COL7A1 knockdown in CAFs co-cultured with in BxPC-3 cells. * p<0.05; ** p<0.01; *** p<0.001; **** p<0.0001.

PC cells (PANC-1, BxPC-3) and CAFs were resuspended to a concentration of 1×10^6^ cells/mL in DMEM complete medium. The PC cells and CAFs were then co-cultured in a 6-well plate transwell system with a pore size of 0.4 μm (Corning, USA) (**Figure 1A**). A total of 200 μl of CAF suspension was added to the upper chamber, and 800 μl of PC cell suspension was seeded into the lower well of the plate.

### qRT-PCR

Total RNA was extracted from CAFs using TRIzol Reagent (15596026; Ambion, Life Technologies, Carlsbad, CA, USA), followed by first-strand cDNA synthesis using a First-Strand Synthesis System for qRT-PCR (A6001, Promega, Madison, USA). The resulting cDNA was quantified by real-time PCR on a Veriti 96-well Thermal Cycler (4375786; Applied Biosystems, Foster City, CA, USA). Amplification was performed using a StepOnePlus™ Real-Time PCR System (Applied Biosystems) according to the manufacturer’s instructions. The forward primer sequence for COL7A1 is 5’-GTTGGAGAGAAAGGTGACGAGG-3’, and the reverse primer sequence is 3’-TGGTCTCCCTTTTCACCCACAG-5’. GAPDH was used as the internal reference gene, with the following primer sequences: forward primer 5’-GTCTCCTCTGACTTCAACAGCG-3’, reverse primer 3’-ACCACCCTGTTGCTGTAGCCAA-5’. Relative expression levels were normalized to GAPDH and calculated using the -2^ΔΔ^CT method.

### Western blot analyses

The detailed procedures of western blot analyses were analogous to our previous study (35). The primary antibodies employed were as follows: rabbit anti-COL7A1 (1:600 dilution; 19799-1-AP, Proteintech, Chicago, IL, USA) and rabbit anti-β-actin (1:1000 dilution; ab8226, Abcam, Cambridge, UK).

### Wound healing assay

The processed cells were seeded in 6-well plates at a density of 3×10^5^ cells/well in FBS-free medium. When cells reached 70-80% confluence, sterile pipette tips were used to scratch and form a “wound”. The cells were then incubated for 24 hours, after which wound closure was observed and images were captured using a DFC300FX microscope (Leica, Jena, Germany). The width of the wound and the number of migrated cells were quantified using Image J software (NIH, Bethesda, MD, USA).

### Transwell assay

PC cells were seeded in the upper chambers of a transwell system (pore size: 8 μm; Corning, USA) at a density of 2×10^5^ cells per well in 200 μl of serum-free DMEM, whereas CAFs were cultured in the lower chambers at a density of 1×10^6^ cells per well in 800 μl of complete DMEM. Following 24 hours of incubation, the cells were fixed and stained with crystal violet for 15 mins. Cell migration was assessed by capturing images under a microscope and counting cells in five randomly selected fields of view. The cell number was further obtained using Image J software (NIH, Bethesda, MD, USA).

### Colony formation assay

500 preprocessed PC cells were seeded into each well of a 6-well plate and cultured in a cell incubator for 14 days. Following this, the cells were fixed with 4% paraformaldehyde for 20 minutes and subsequently stained with crystal violet for 10 minutes. After staining, the excess dye was removed by washing three times with phosphate-buffered saline (PBS). The resulting colonies were photographed and quantified using Image J software (NIH, Bethesda, MD, USA).

### Cell viability assay

4×10^3^ preprocessed PC cells were seeded into 96-well plates and incubated for 8 h prior to treatment with various concentrations of gemcitabine (GEM) (LILLY, France): 0, 1 nM, 10 nM, 100 nM, 1 μM, 10 μM, 100 μM, and 1 M. After 48 h of drug treatment, CCK-8 kit (Dojindo, Japan) was used to detect cell viability according to the manufacturer’s instructions. Absorbance was measured at 562 nm. Dose-response curves were generated by nonlinear regression analysis (inhibitor, four parameters) using GraphPad Prism 10, and the half-maximal inhibitory concentration (IC50) was determined as the drug concentration corresponding to the steepest slope of the fitted curve.

### Statistical analysis

All statistical analyses were performed using R software (version 4.4.1). The Student’s t test was used to compare means between two groups. The OS of patients in high- and low-risk groups was compared using Kaplan-Meier analysis with the log-rank test. Independent predictors for patient prognosis were identified through univariate and multivariate Cox regression analyses. The Wilcoxon rank-sum test was applied to compare gene expression levels, as well as variations in TMB, drug sensitivity, immune scores, TIDE scores, and immunohistochemistry positive score (IPS) between the two risk groups. Spearman correlation analysis was used to assess the relationship between these variables. All analyses were systematically repeated to ensure result reliability. Two-tailed p-values less than 0.05 were considered statistically significant.

## Results

### Identification of PCLM and BM-related genes

**Figure 2:**
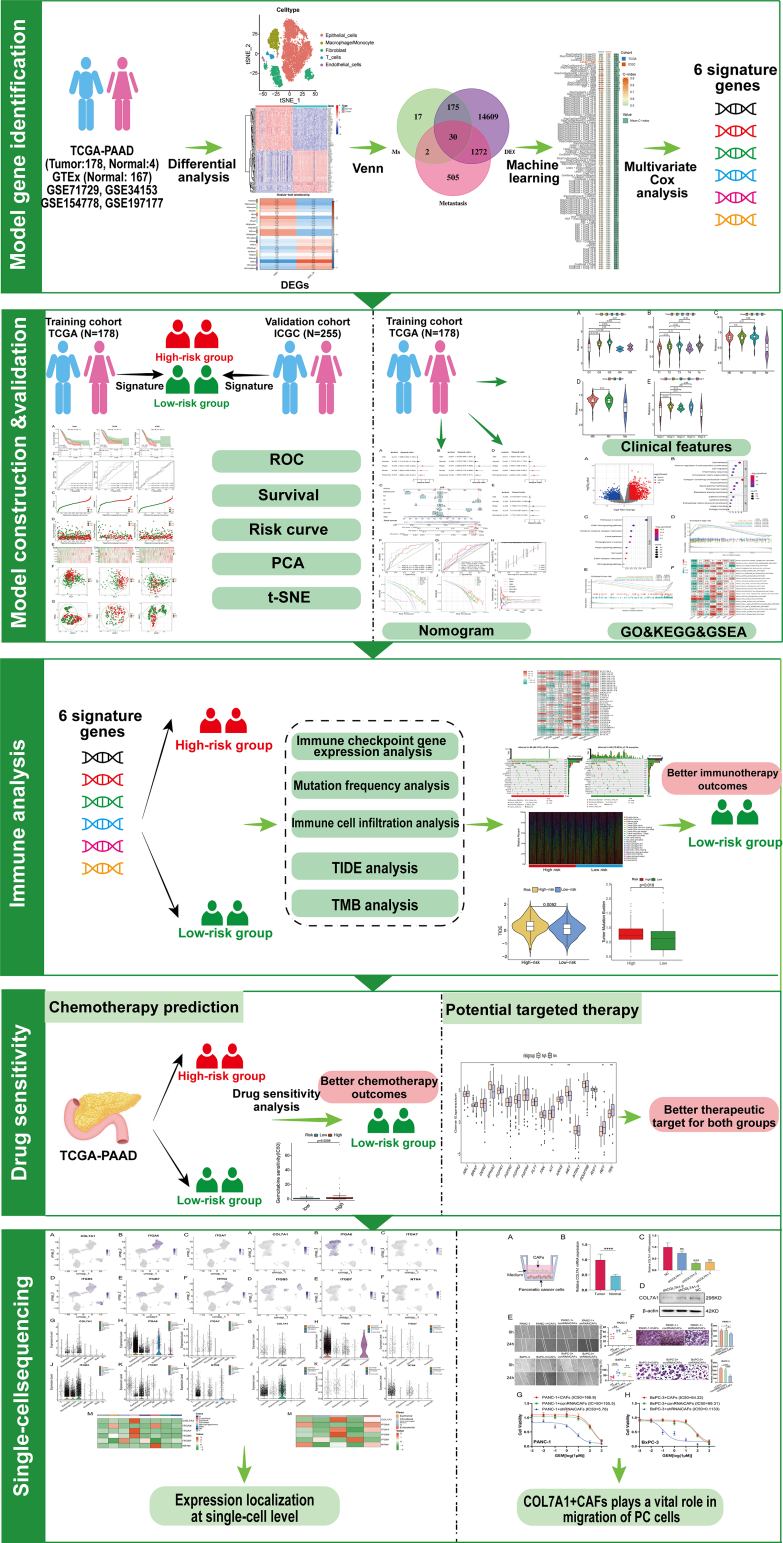
Workflow chart of the study. GO, Gene Ontology; GSEA, gene set enrichment analysis; ICGC, International Cancer Genome Consortium; KEGG, Kyoto Encyclopedia of Genes and Genomes; PCA, principal component analysis; ROC, receiver operating characteristic; TCGA, The Cancer Genome Atlas; TIDE, tumor immune dysfunction and exclusion; TMB, tumor mutation burden; t-SNE, t-distributed stochastic neighbor embedding.

**Figure 3:**
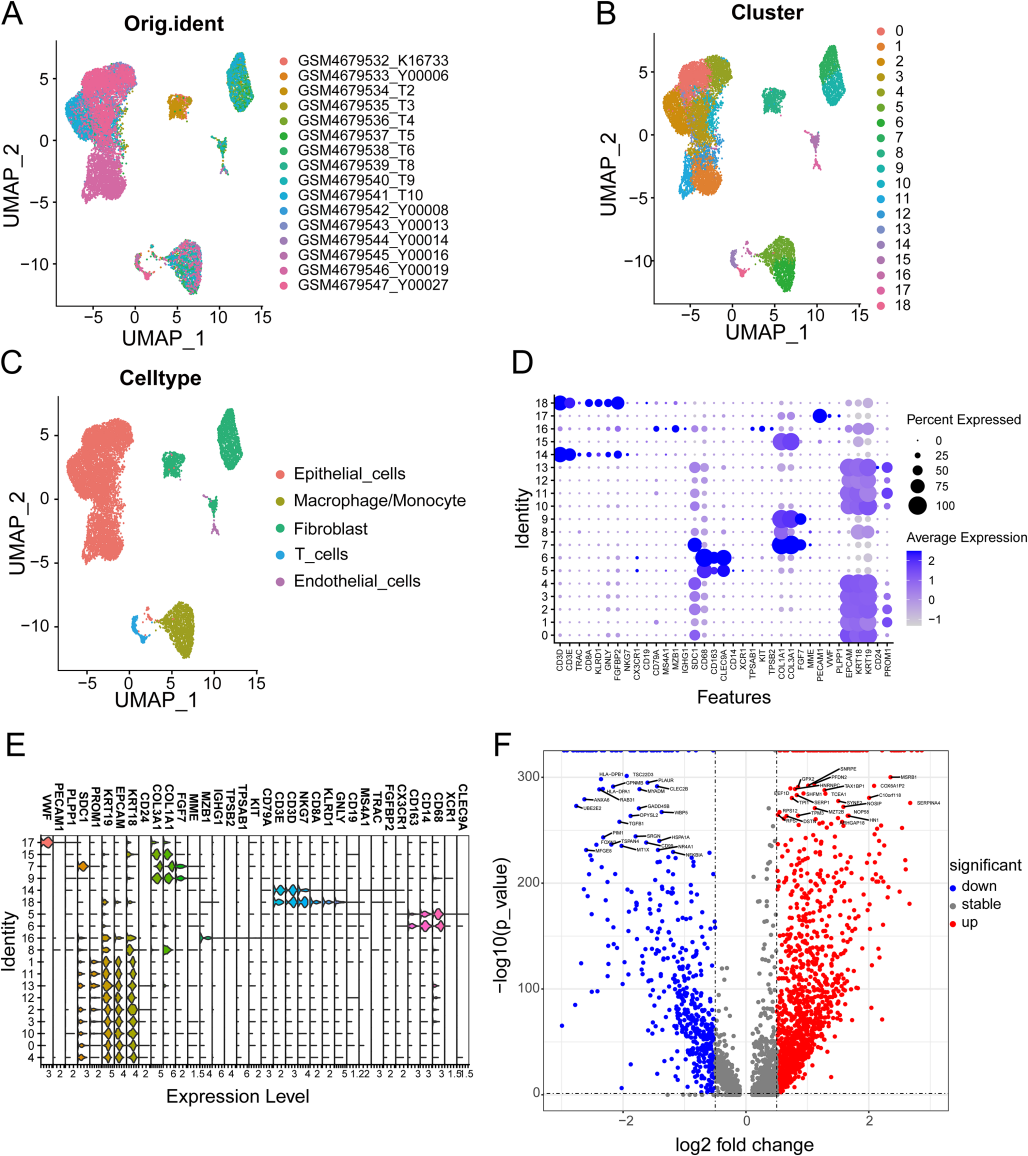
**(A)** The original UMAP plot of GSE154778; **(B)** The UMAP plot of GSE154778 after simple annotation by the SingleR R package; **(C)** The UMAP plot of GSE154778 after manual marker annotation; **(D)** Dot plot of expression levels of each cell population in manual annotation in GSE154778; **(E)** Violin plot of expression levels of each cell population in manual annotation in GSE154778; **(F)** Volcano plot of GSE154778, showing significantly upregulated and downregulated differentially expressed genes (DEGs).

**Figure 4:**
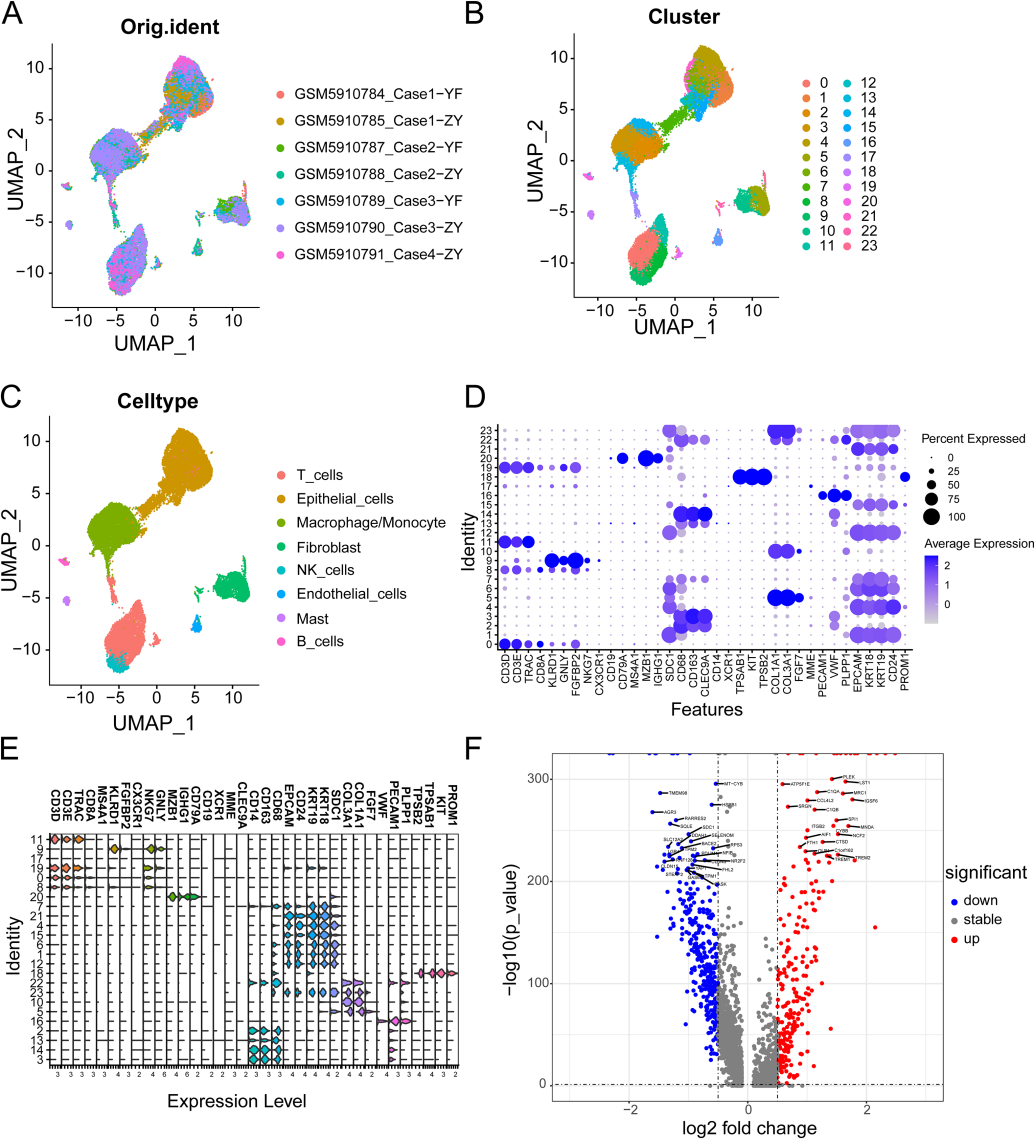
**(A)** The original UMAP plot of GSE197177; **(B)** The UMAP plot of GSE197177 after simple annotation by the SingleR R package; **(C)** The UMAP plot of GSE197177 after manual marker annotation; **(D)** Dot plot of expression levels of each cell population in manual annotation in GSE197177; **(E)** Violin plot of expression levels of each cell population in manual annotation in GSE154778; **(F)** Volcano plot of GSE197177, showing significantly upregulated and downregulated differentially expressed genes (DEGs).

**Figure 5:**
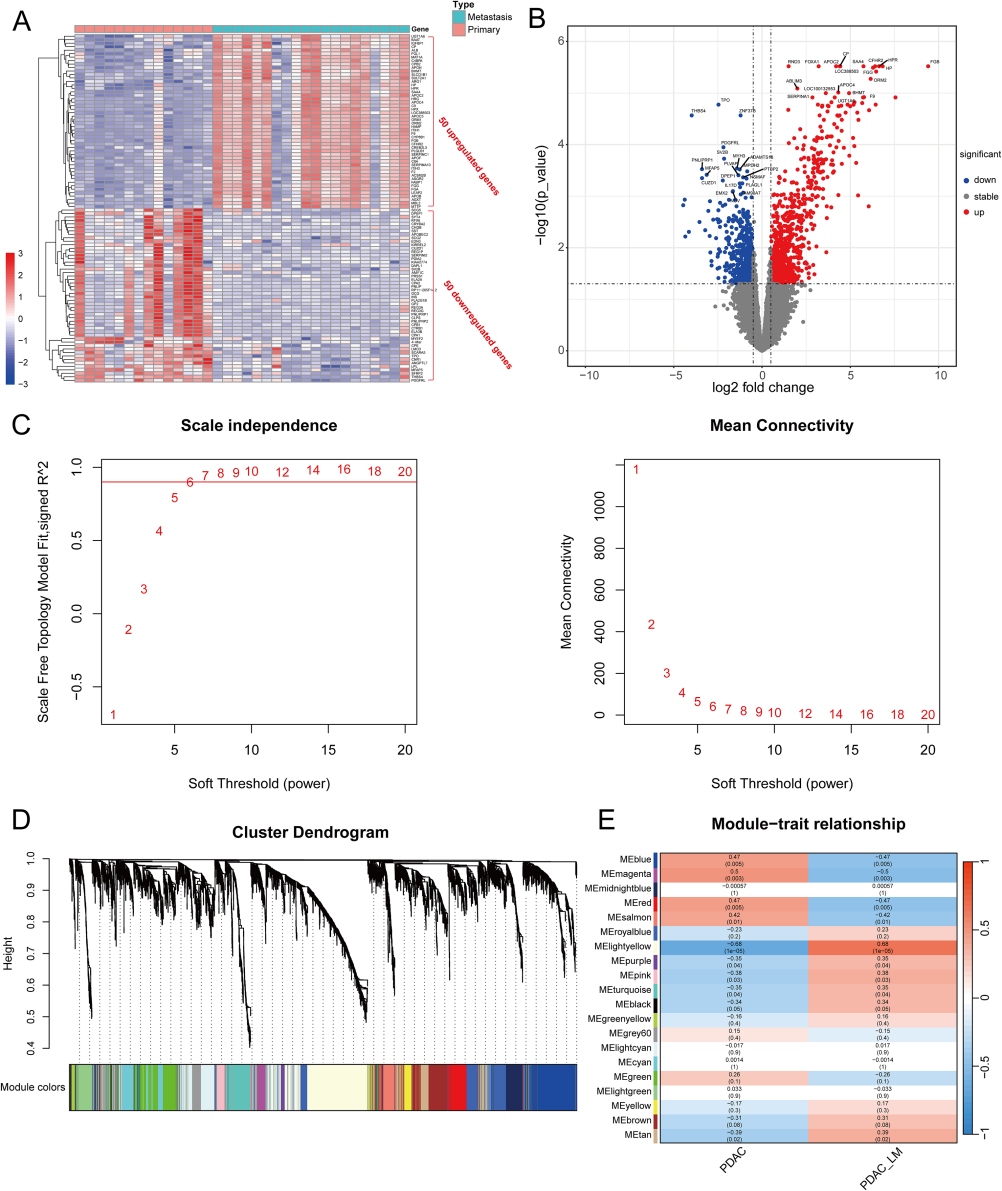
**(A)** Heatmap of the top 50 upregulated and downregulated DEGs between primary tumors and metastases in GSE34153; **(B)** Volcano plot of DEGs between primary tumors and metastases in GSE34153; **(C)** Selecting the optimal β parameter for WGCNA analysis in GSE34153. **(D)** System tree diagram of gene set clusters from WGCNA analysis; **(E)** Module diagram of co-expressed genes from WGCNA analysis.

**Figure 6:**
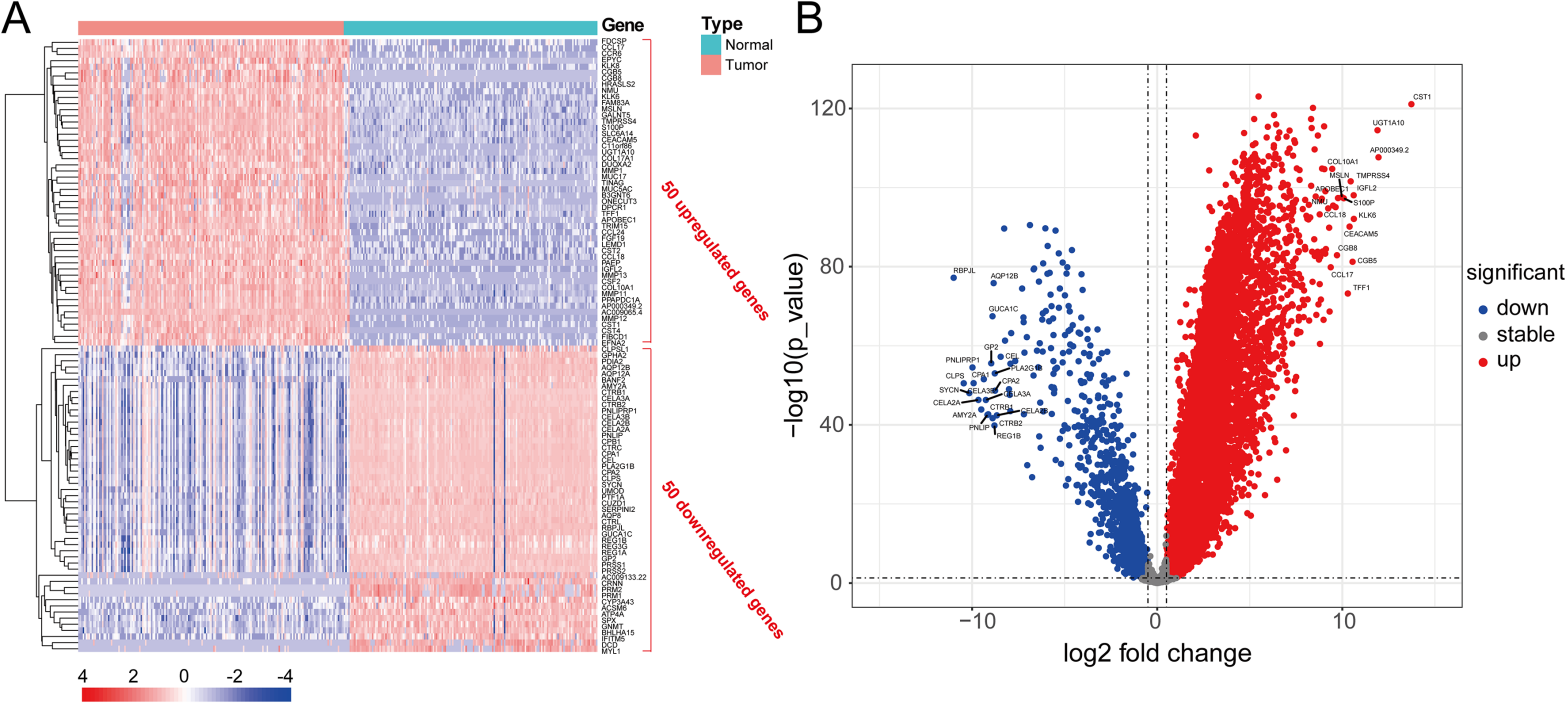
**(A)** Heatmap of the top 50 significantly upregulated and downregulated DEGs in pancreatic cancer and adjacent tissues analyzed by the TCGA and GTEx databases; **(B)** Volcano plot of significantly DEGs in pancreatic cancer and adjacent tissues analyzed by the TCGA and GTEx databases.

**Figure 7:**
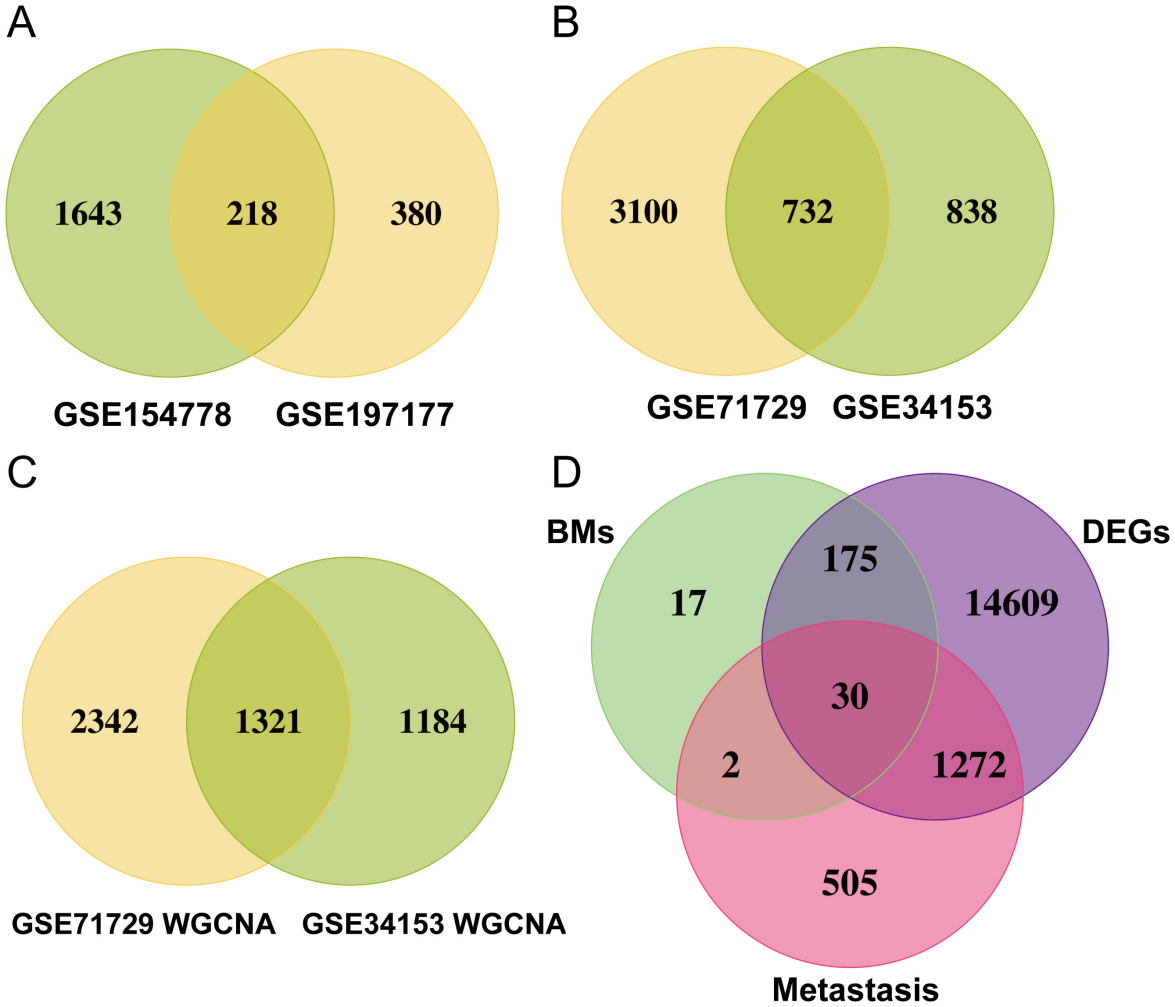
**(A)** Venn diagram of significantly DEGs in the GSE154778 and GSE197177 datasets; **(B)** Venn diagram of significantly DEGs in the GSE71729 and GSE34153 datasets; **(C)** Venn diagram of significantly DEGs in the WGCNA analysis of the GSE71729 and GSE34153 datasets; **(D)** Venn diagram showing 30 common genes selected.

**Figure 2** shows the workflow chart of the study. Both single-cell and bulk transcriptional data were utilized to identify PCLM-related genes. Using the Seurat R package (Version 5.1.0), we analyzed the single-cell transcriptional datasets GSE154778 and GSE197177, employing UMAP and tSNE methods to visualize cell clustering. In GSE154778, we included 10 primary tumor samples and 6 metastatic tumor samples, whereas in GSE197177, 3 paired primary and metastatic tumor samples were incorporated (**Figures 3A**, **4A** and **Supplementary Figures S1A, D**). The SingleR R package was used to divide the cells into 19 clusters for GSE154778 and 24 clusters for GSE197177, respectively (**Figures 3B**, **4B** and **Supplementary Figures S1B, E**). Through manual annotation, five cell types were identified in the GSE154778 cohort and eight cell types in the GSE197177 cohort (**Figures 3C**, **4C** and **Supplementary Figures S1C, F**). **Figures 3D, E** illustrate the expression of classical markers across different cell clusters for manual annotation in GSE154778, while **Figures 4D, E** present the corresponding results for GSE197177. Subsequently, we identified significantly DEGs between primary and metastatic tumor samples in both datasets using the criteria log2 |Fold change| ≥ 0.5 and FDR < 0.05. A total of 1861 DEGs were detected in GSE154778 and 598 in GSE197177, with the volcano maps displayed in **Figures 3F** and **4F** for the respective cohorts. The GSE34153 and GSE71729 cohorts were recruited to identify DEGs between primary and metastatic tumors and to perform WGCNA analysis. In the WGCNA, the optimal β parameters for achieving scale-free topology were determined to be 6 and 7 for GSE34153 and GSE71729, respectively. Subsequently, a total of 20 and 21 co-expression modules were identified in GSE34153 and GSE71729, respectively. The results showed that 1570 DEGs were detected in the GSE34153 cohort, along with 1184 liver metastasis-associated co-expression genes derived from WGCNA (**Figure 5**). In the GSE71729 cohort, 3832 DEGs were identified, and 2342 co-expression genes were obtained through WGCNA (**Supplementary Figure S2**). Subsequently, we retrieved 349 TCGA-PAAD & GTEx Pancreas patient samples (178 tumor and 171 normal) encompassing 19726 genes from both the TCGA and GTEx databases. Differential expression analysis was then conducted using the screening criteria of log2 |Fold change| ≥ 0.5 and FDR < 0.05, yielding a total of 16086 DEGs. **Figure 6A** displays the heatmap of the top 50 positively and negatively expressed DEGs between PC and normal pancreatic tissues, while **Figure 6B** demonstrated the volcano maps of the DEGs. Subsequently, we identified the intersection of DEGs in GSE154778 and GSE197177, resulting in 218 common DEGs (**Figure 7A**). Moreover, we found 732 common DEGs between GSE34153 and GSE71729, as well as 1321 PCLM-related genes through WGCNA (**Figures 7B, C**). Finally, by intersecting 224 BM-related genes, 1809 PCLM-related genes, and 16085 PC-related DEGs, we obtained 30 signature genes for subsequent analysis (**Figure 7D**).

### Machine learning based integration develops a novel prognostic model for PC

**Figure 8:**
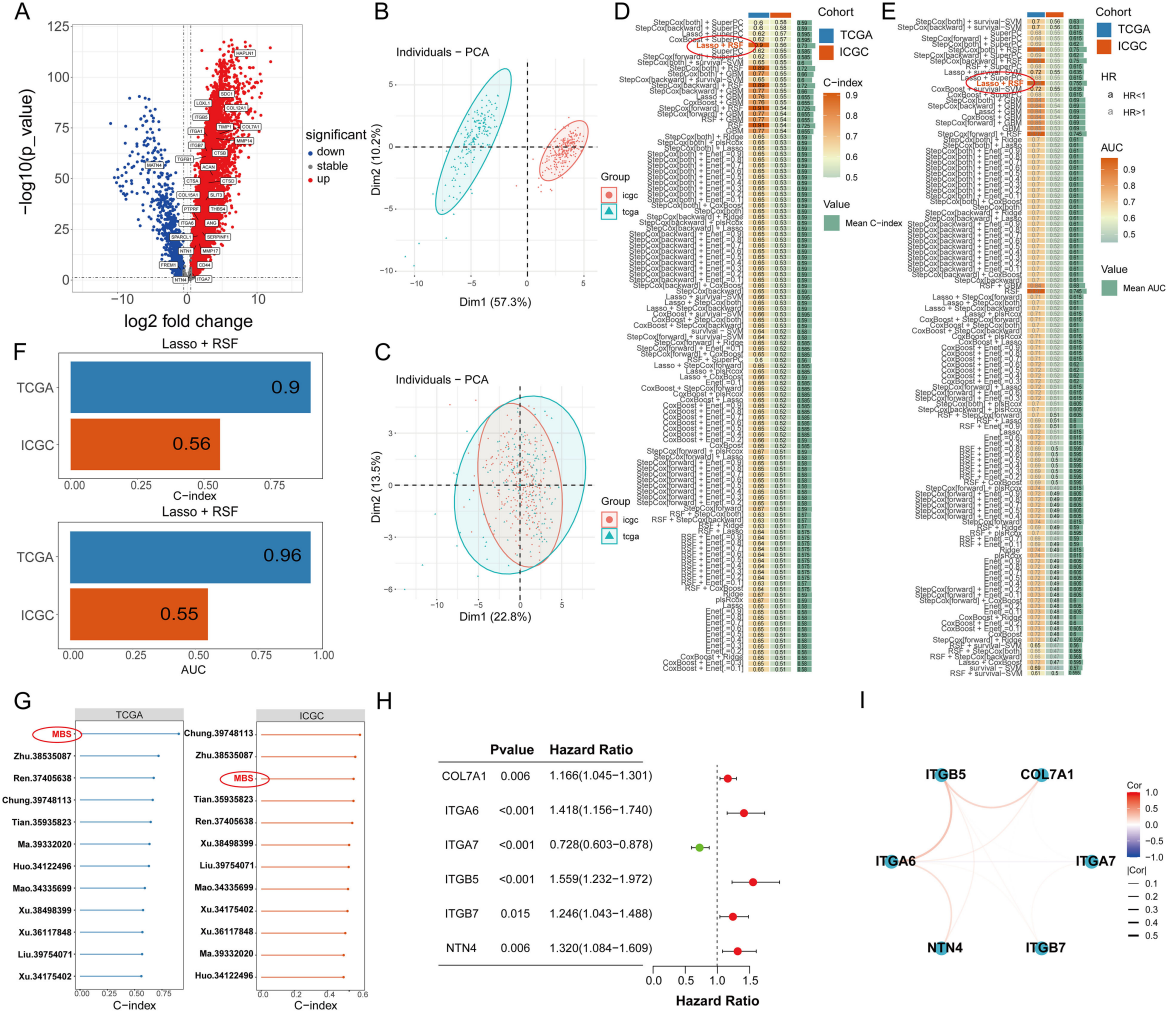
**(A)** The expression of 30 PCLM and BM-related genes in a heat map. **(B)** PCA plot of training and validation groups before removing batch effect. **(C)** PCA plot of training and validation groups after removing batch effect. **(D)** The C-index values of the training set and validation set for each of the 100 machine learning prediction models were calculated. **(E)** The ROC values of the training set and validation set for each of the 100 machine learning prediction models were calculated. **(F)** C-index value of Lasso+RSF model in TCGA and ICGC cohort. **(G)** Forest plots present the C-index values of our model and previous models in training and validation models. **(H)** A forest plot shows the prognostic value of 6 related genes in pancreatic cancer. **(I)** A gene correlation network map shows the expression correlation among six genes in the prognostic model.

**Figure 9:**
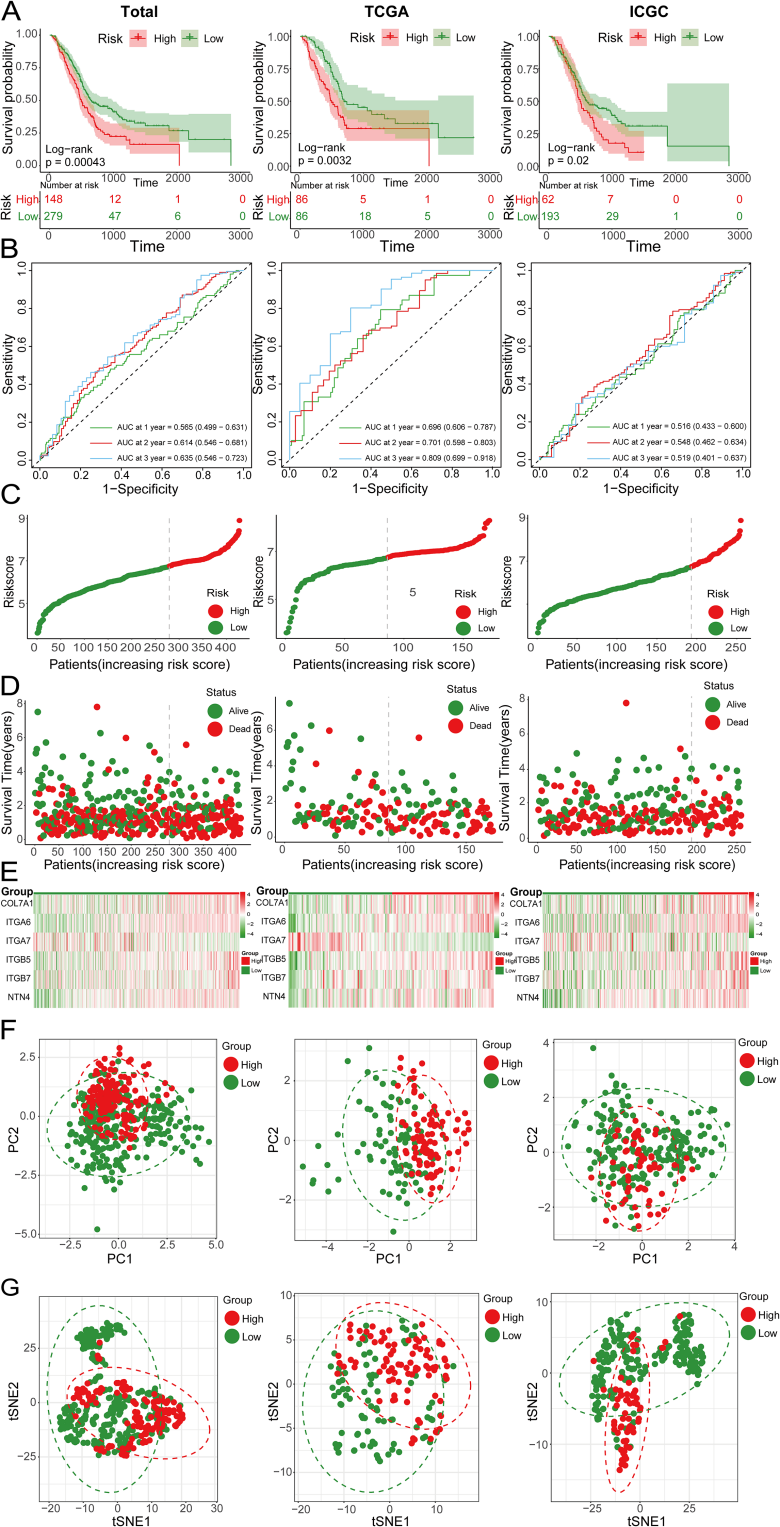
**(A)** Kaplan-Meier survival curves showing the OS of high-risk and low-risk groups in the total, TCGA and ICGC cohorts; **(B)** ROC curves of the total, TCGA and ICGC cohorts; **(C)** Risk score curves of the total, TCGA and ICGC cohorts; **(D)** Scatter plots of survival status in the total, TCGA and ICGC cohorts; **(E)** Heatmap showing the expression levels of 6 genes in high-risk and low-risk groups; **(F)** PCA plots of the total, TCGA and ICGC cohorts; **(G)** tSNE plots of the total, TCGA and ICGC cohorts.

Initially, we meticulously annotated 30 PCLM and BM-related genes obtained from our prior analysis within the heatmap of significantly DEGs between PC and adjacent normal pancreatic tissues. Among these genes, except for MATN4 and FREM1, the expression levels of the remaining genes were markedly upregulated in PC compared to adjacent normal tissues (**Figure 8A**). Subsequently, we utilized data from the TCGA database as the training set and data from the ICGC database as the validation set. To ensure data consistency, batch effects were removed from both datasets. Comparisons of PCA plots before and after batch effect removal demonstrated that the removal process effectively minimized technical variation (**Figures 8B, C**). The leave-one-out cross-validation (LOOCV) framework was employed to optimize a combination of 10 machine learning algorithms with hyperparameter tuning using the training set. Thereafter, the C-index and AUC values for each model were calculated using the validation set. The optimal model was identified as the combination of Lasso and Random Survival Forest (RSF), which achieved the highest average C-index (0.73) and AUC (0.755) among all model types (**Figures 8D, E**). **Figure 8F** displays the detailed C-index and AUC values of the Lasso+RSF model in both the training and validation datasets. Compared with previously reported prognostic models, the model developed in this study demonstrated superior performance in terms of efficacy across both the TCGA and ICGC databases (**Figure 8G**) (36–46). Six consensus genes with prominent prognostic value were identified, and their gene coefficients were further calculated in the model. Univariate analysis revealed that all six genes exhibited significant prognostic value (p<0.05). Specifically, except for ITGA7, increased expression levels of the remaining five genes were significantly associated with poorer prognosis in PC patients (**Figure 8H**). The gene correlation network diagram indicates that, apart from ITGA7, the expression levels of the other five genes display significant positive correlations with one another (**Figure 8I**). Subsequently, multivariate COX regression analysis was conducted on these six genes to determine the coefficients for each prognostic gene. Based on these coefficients, we formulated a prognostic scoring model as follows: Risk Score = (COL7A1 * 0.126) + (ITGA6 * 0.182) - (ITGA7 * 0.271) + (ITGB5 * 0.346) + (ITGB7 * 0.344) + (NTN4 * 0.222). The median value of the prognostic risk scores in the training dataset was used as the cut-off point to classify patients. We then stratified PC patients in both the training set (TCGA dataset) and the validation set (ICGC dataset) into high-risk and low-risk groups. Kaplan-Meier survival curves for the overall cohort and the two sub-cohorts consistently demonstrated that the high-risk group exhibited significantly reduced survival rates compared to the low-risk group (**Figure 9A**). Furthermore, the ROC curves confirmed the robust prognostic performance of this model at 1-, 2-, and 3-year OS, as evidenced by the relatively high AUC values (**Figure 9B**). Additional analysis of the risk curve and survival status also revealed that patients in the high-risk group had markedly shortened OS. Moreover, the expression levels of the five genes (COL7A1, ITGA6, ITGB5, ITGB7, NTN4) were significantly elevated in the high-risk group compared to the low-risk group (**Figures 9C-E**). PCA and tSNE plots clearly demonstrated that the two risk groups were distinctly separated, forming two separate clusters (**Figures 9F, G**). Moreover, the bar chart indicated that the expression levels of COL7A1, ITGA6, ITGB5, ITGB7, and NTN4 were significantly higher in the high-risk group than in the low-risk group. In contrast, ITGA7 showed a markedly reduced expression level in the high-risk group (**Supplementary Figure S3**). Additionally, survival analysis revealed that elevated expression levels of the prognostic genes, except for ITGA7, were significantly associated with worse patient outcomes. Conversely, increased expression of ITGA7 was significantly correlated with better patient prognosis (**Supplementary Figure S4**). In addition, we explored the correlation between risk scores and clinicalpathological variables. In the TCGA dataset, this risk score was significantly associated with the tumor stage of PC patients, whereas in the ICGC dataset, it was strongly correlated with tumor size. However, this prognostic model showed no significant correlation with the TNM stage of the tumor in either dataset (**Supplementary Figure S5**). This could potentially be attributed to the heterogeneity between the two datasets.

### Validation and evaluation of the novel model and the gene set enrichment analyses

**Figure 10:**
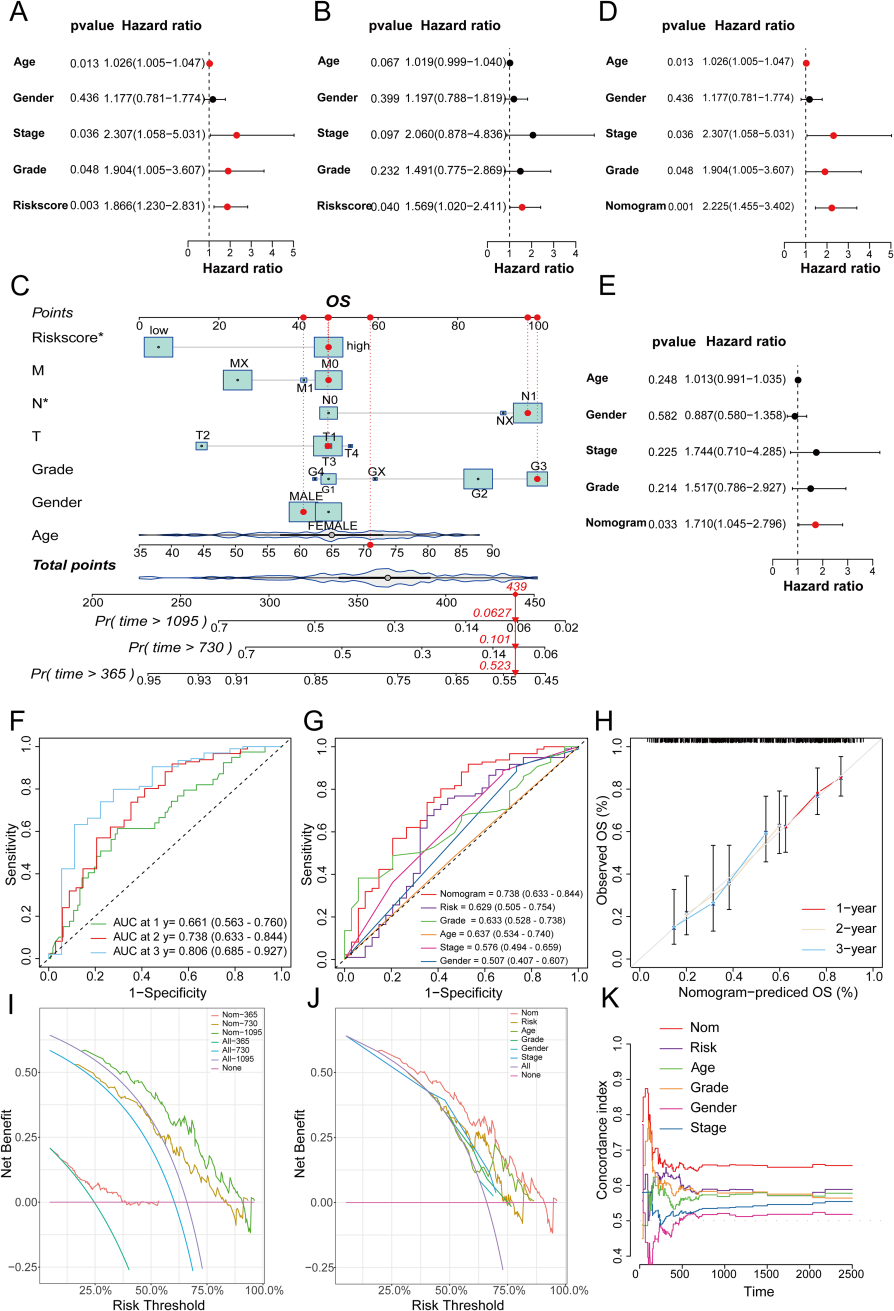
**(A)** Univariate analysis of the TCGA cohort; **(B)** Multivariate analysis of the TCGA cohort; **(C)** Nomogram for constructing a combined clinical variable and prognostic model risk score in the TCGA dataset; **(D)** Univariate analysis of the TCGA cohort, including the nomogram model; **(E)** Multivariate analysis of the TCGA dataset, including the nomogram model; **(F)** ROC curve prediction model for 1-, 2-, and 3-year survival prediction accuracy in the TCGA cohort; **(G)** Multivariate ROC curves to plot the prognostic prediction efficacy of gender, age, stage, grade, the risk score of the prognostic model, and the nomogram; **(H)** 1-, 2-, and 3-year calibration curves to evaluate the prognostic prediction stability of the nomogram; **(I)** DCA curves to show the clinical benefit level of the nomogram model at 1-, 2-, and 3-years; **(J)** DCA curves to show the corresponding clinical benefits of the nomogram model, the risk score of the prognostic model, and other clinical variables; **(K)** C-index curves to evaluate the prognostic efficacy of the nomogram, the risk score of the prognostic model, and related clinical variables.

**Figure 11:**
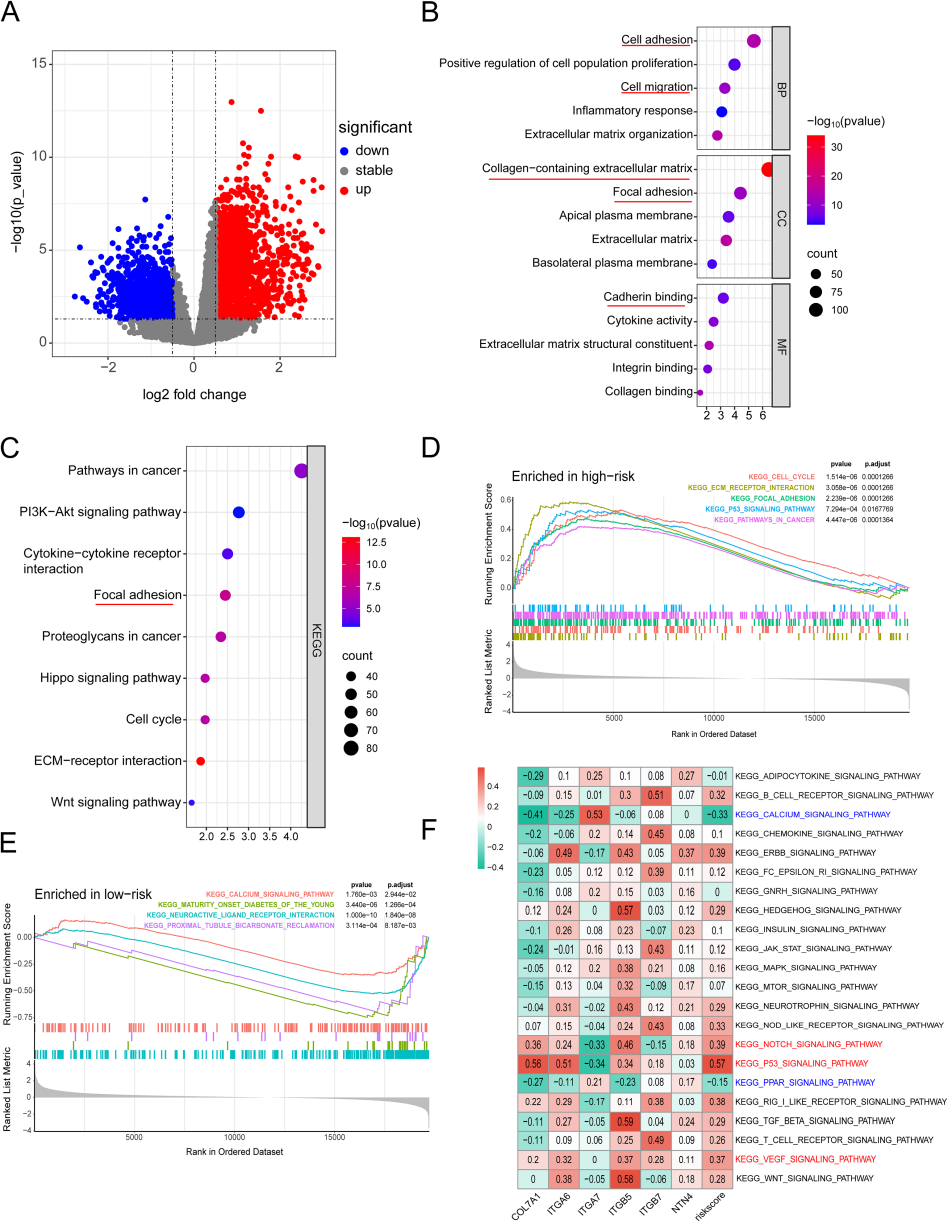
**(A)** Heatmap of significantly DEGs between high-risk and low-risk groups; **(B)** GO enrichment analysis plot; **(C)** KEGG analysis plot; **(D)** Top 5 significantly enriched signaling pathways in the high-risk group; **(E)** Significantly enriched signaling pathways in the low-risk group; **(F)** GSVA analysis demonstrating the correlation between tumor-related KEGG signaling pathways and model genes.

To further validate the prognostic value of the predictive model we constructed and compare its prognostic performance with other clinicalpathological variables, we performed univariate and multivariate analyses using the TCGA dataset. The results demonstrated that age, TNM stage, tumor grade, and the risk score derived from the prognostic model were independent prognostic factors in the univariate analysis (**Figure 10A**). In the subsequent multivariate analysis, only the risk score of the prognostic model remained an independent prognostic factor (**Figure 10B**). Subsequently, we integrated the risk score of the prognostic model with relevant clinicalpathological variables (age, gender, T stage, N stage, M stage, and tumor grade) to develop a prognostic nomogram (**Figure 10C**). Further univariate and multivariate analysis confirmed that the nomogram was an independent prognostic factor in both univariate and multivariate analyses (**Figures 10D, E**). The subsequent ROC analysis revealed that the nomogram exhibited superior prognostic performance in predicting 1-, 2-, and 3-year OS, with an AUC higher than that of the 6-gene prognostic model alone. Additionally, its prognostic performance surpassed that of any single clinicalpathological variable (**Figures 10F, G**). The subsequent calibration curves, DCA, and time-dependent C-index curves all indicated that the nomogram had excellent prognostic predictive accuracy (**Figures 10H-K**). In the ICGC dataset, while the overall results were less satisfactory compared to the training set, similar trends were observed. This discrepancy might be attributed to sample heterogeneity, treatment differences and the relatively small size of training cohort (**Supplementary Figure S6**). To further explore the differences in signaling pathways between the high and low-risk groups, we identified significantly DEGs between these two groups. Specifically, a total of 2020 upregulated genes and 1098 downregulated genes were detected in the high-risk group (**Figure 11A**). These DEGs were subsequently subjected to GO and KEGG enrichment analyses using the Database for Annotation, Visualization, and Integrated Discovery (DAVID). The GO analysis indicated that the DEGs were significantly enriched in biological processes and molecular functions associated with cell adhesion, migration, focal adhesion, integrin binding, and collagen-containing extracellular matrix, all of which are closely linked to cell invasion and metastasis (**Figure 11B**). KEGG analysis revealed that the DEGs were significantly associated with tumor-related signaling pathways, including the PI3K-Akt signaling pathway, Hippo signaling pathway, Wnt signaling pathway, and extracellular matrix (ECM)-receptor interaction (**Figure 11C**). Furthermore, GSEA demonstrated that in the high-risk group, these genes were significantly enriched in pathways related to the cell cycle, ECM-receptor interaction, focal adhesion, p53 signaling, and pathways in cancer (**Figure 11D**). In contrast, the low-risk group exhibited no significant enrichment in pathways associated with tumor progression (**Figure 11E**). Finally, GSVA showed that the genes in the prognostic model were positively associated with the majority of signaling pathways implicated in tumor progression. Additionally, the risk score was significantly positively correlated with the p53 signaling pathway, Notch signaling pathway, and VEGF signaling pathway, while it was significantly negatively correlated with the PPAR signaling pathway and calcium signaling pathway (**Figure 11F**, **Supplementary Figure S7**).

### Comparison of tumor mutation burden and drug sensitivity between high and low-risk groups

**Figure 12:**
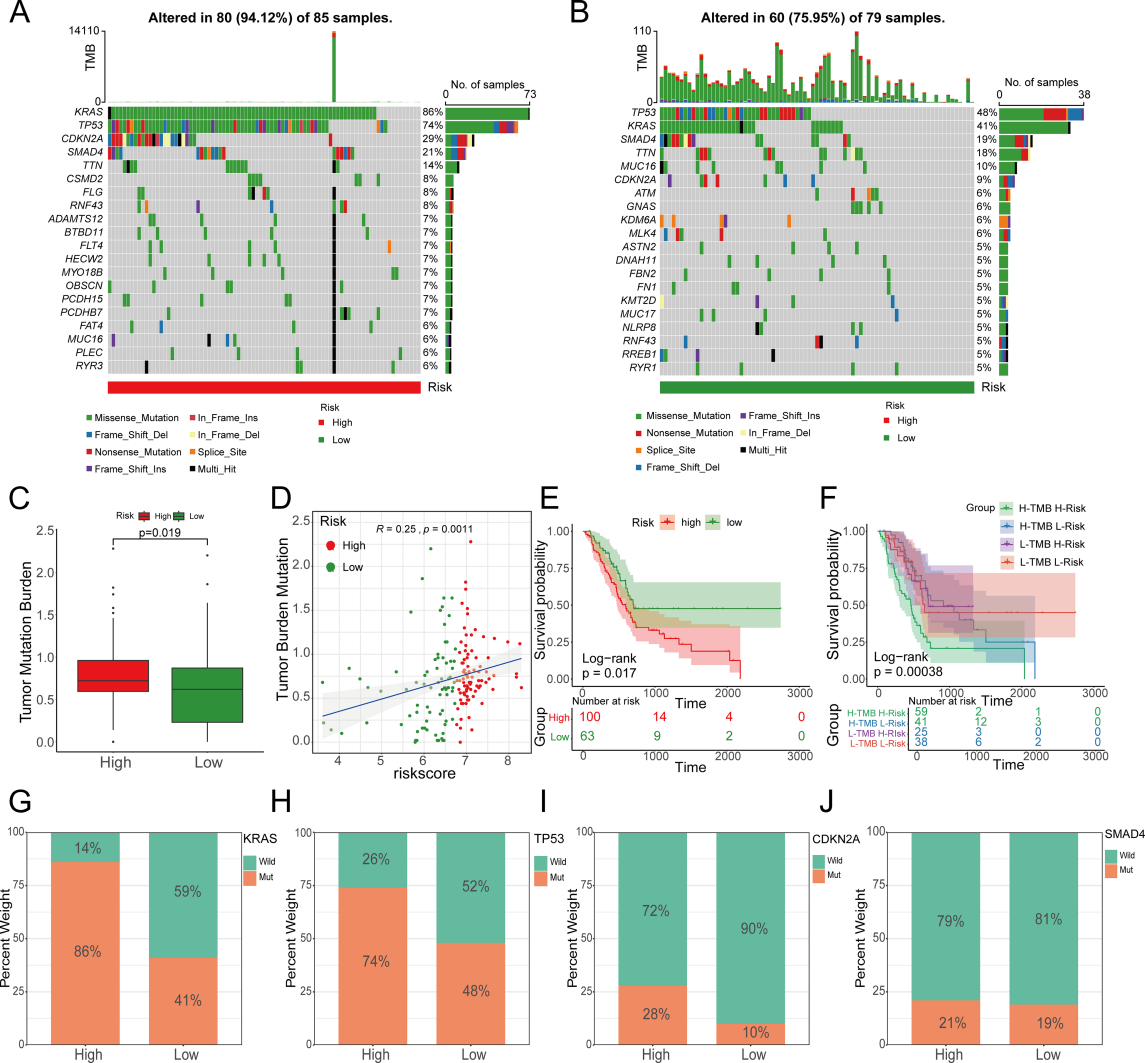
The relationship between the prognostic model risk score and TMB. **A, (B)** The top 20 mutated genes in the high- and low-risk groups; **(C)** TMB in the high-risk group was significantly higher than that in the low-risk group; **(D)** The risk score was significantly correlated with TMB; **(E, F)** TMB and risk score were significantly associated with poor prognosis. **(G)** KRAS, **(H)** TP53, **(I)** CDKN2A and **(J)** SMAD4, as the four genes with the highest mutation rates in pancreatic cancer, showed significant changes in the high- and low-risk groups.

**Figure 13:**
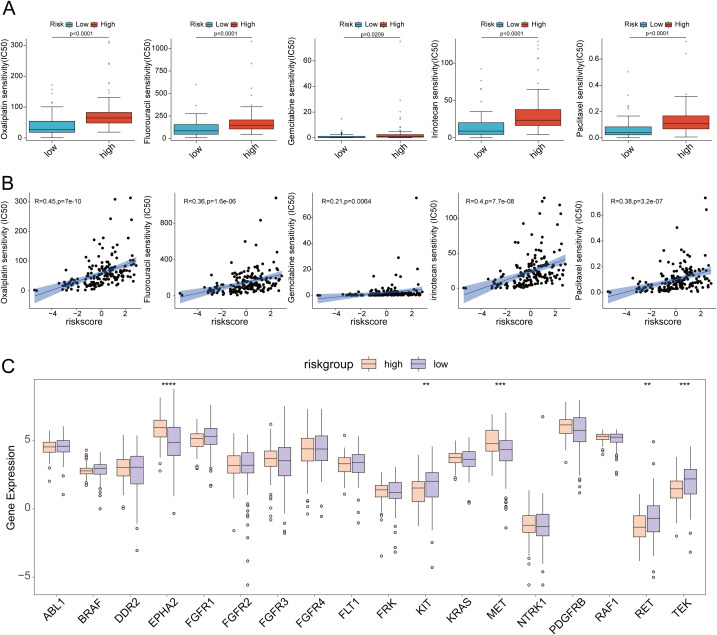
**(A)** Drug sensitivity analysis of oxaliplatin, fluorouracil, gemcitabine, irinotecan and paclitaxel in high-risk and low-risk groups; **(B)** Scatter plot of drug sensitivity analysis of oxaliplatin, fluorouracil, gemcitabine, irinotecan and paclitaxel in high-risk and low-risk groups; **(C)** Differences in target gene expression between high-risk and low-risk groups. ** p<0.01; *** p<0.001; **** p<0.0001.

Mutations in several key genes are among the critical contributors to PC development. Subsequently, we analyzed the tumor mutational profiles of patients in both the high- and low-risk groups. The somatic mutation burden analysis revealed that 94.12% of patients in the high-risk group harbored gene mutations, significantly higher than the 75.95% mutation rate observed in the low-risk group. Notably, the most frequent mutation types included missense mutations, nonsense mutations, and frameshift deletions (**Figures 12A, B**). A significant positive correlation was identified between the risk score and tumor mutational burden (TMB) (R = 0.25, p=0.0011). Moreover, TMB levels were significantly elevated in the high-risk group compared to the low-risk group (**Figures 12C, D**). Survival analysis integrating TMB and risk score demonstrated that patients with low TMB had a significantly better prognosis than those with high TMB. Importantly, patients with both low TMB and low risk scores exhibited the most favorable survival outcomes (**Figures 12E, F**). In the high-risk group, the mutation frequencies of canonical oncogenes KRAS, TP53, and CDKN2A were significantly higher than those in the low-risk group. Conversely, no significant difference was observed in the mutation frequency of the tumor suppressor gene SMAD4 between the two groups in our study (**Figures 12G-J**). Subsequently, we examined the chemotherapeutic sensitivity of PC patients across different risk groups. Using the OncoPredict R package, we assessed the efficacy differences of commonly used PC chemotherapeutics between high- and low-risk patients. The results demonstrated that the half-maximal inhibitory concentration (IC50) values for oxaliplatin, fluorouracil, gemcitabine, irinotecan, and paclitaxel were significantly higher in the high-risk group compared to the low-risk group, suggesting that high-risk patients may be more prone to developing chemotherapy resistance (**Figure 13A**). Additionally, correlation scatter plots revealed a significant positive correlation between the risk scores of these drugs and their respective IC50 values (**Figure 13B**). Furthermore, the analysis of expression levels for several common target genes indicated that EPHA2 and MET were significantly upregulated in the high-risk group, whereas KIT, RET, and TEK exhibited significant upregulation in the low-risk group. These findings suggest that targeted therapeutic drugs against these gene targets may exhibit differential efficacy among patients in different risk groups (**Figure 13C**).

### Analysis of the tumor immune landscape in high- and low-risk groups

**Figure 14:**
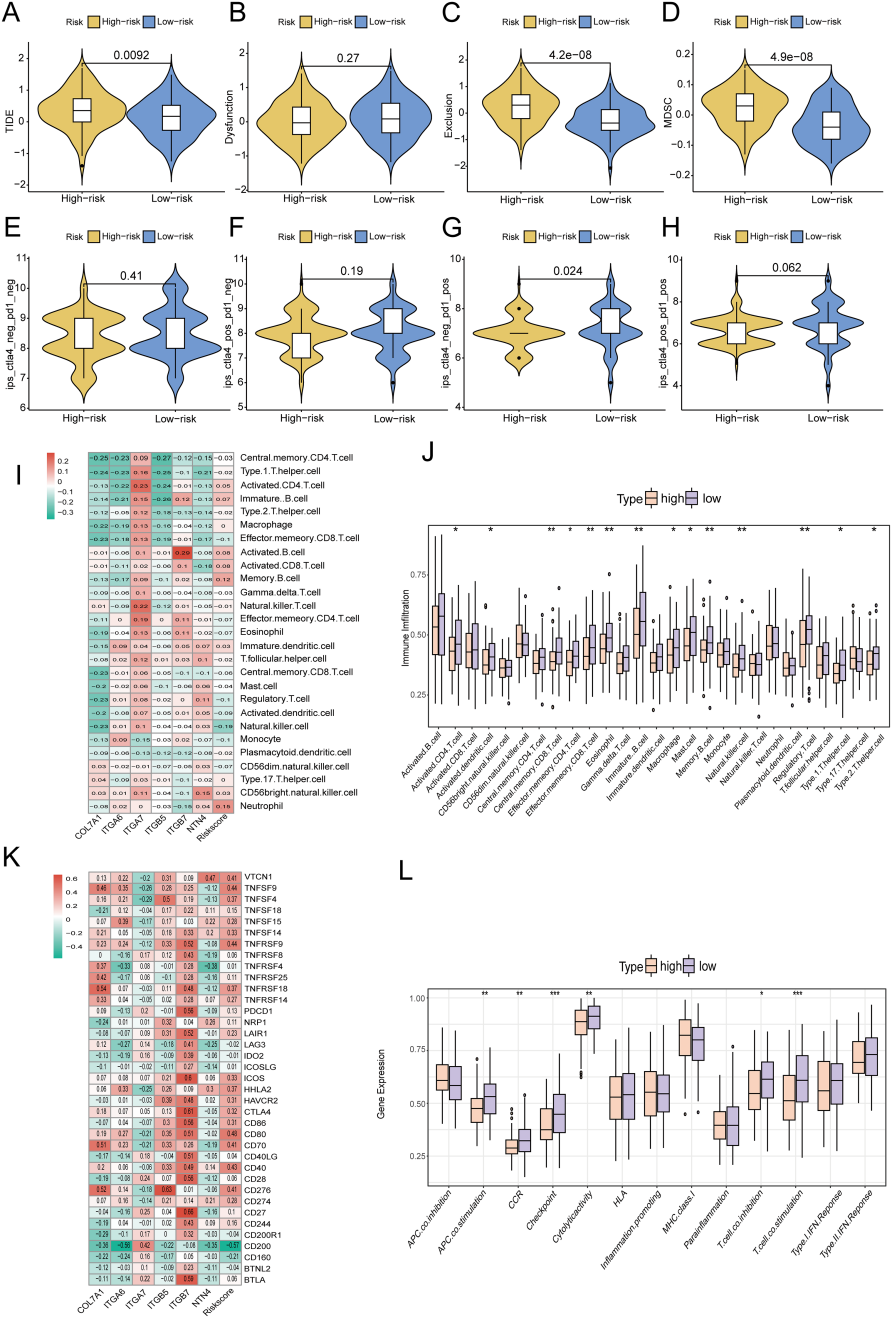
Differences in **(A)** TIDE score; **(B)** Immune dysfunction score; **(C)** Immune rejection score; **(D)** MDSC score between high-risk and low-risk groups; **(E-H)** IPS scores under different expressions of PD1 and CTLA4 in high-risk and low-risk groups; **(I, J)** ssGSEA analysis of differences in immune cell infiltration between high-risk and low-risk groups; **(K)** Heatmap of the correlation between risk score and immune checkpoint gene expression; **(L)** Changes in immune function in high-risk and low-risk groups. * p<0.05; ** p<0.01; *** p<0.001.

The tumor immune microenvironment plays a critical role in tumor progression, invasion, metastasis, and the response to immunotherapy. To evaluate the distinct immune landscapes of high- and low-risk groups, we analyzed differences in TIDE and IPS scores between these groups.The results demonstrated that TIDE scores, immune exclusion scores, and MDSC scores were significantly higher in the high-risk group compared to the low-risk group, indicating a greater likelihood of immune escape and resistance to immunotherapy in the high-risk group (**Figures 14A-D**). In the IPS analysis, the IPS scores for PD1(+)CTLA4(-) were significantly lower in the high-risk group than in the low-risk group. Conversely, no significant differences were observed in the IPS scores for PD1(+)CTLA4(+), PD1(-)CTLA4(-), and PD1(-)CTLA4(+) between the two groups. These findings suggested that the high-risk group exhibited reduced responsiveness to immunotherapy (**Figures 14E-H**). Subsequently, ssGSEA immune cell infiltration analysis revealed that neutrophils and memory B cells exhibited significantly higher infiltration levels in the high-risk group. In contrast, NK cells, CD8+ T cells, γδT cells, and Treg cells demonstrated significantly elevated infiltration levels in the low-risk group (**Figures 14I, J**). Additionally, high-risk scores exhibited significant positive correlations with the expression levels of immune checkpoints such as CD80, TNFSF9, and CD40. Conversely, low-risk scores showed significant positive correlations with the expression of CD200. These findings suggested that the efficacy of different immune checkpoint inhibitors might differ across risk groups (**Figure 14K**). Moreover, immune function analysis revealed that CCR, immune checkpoint expression, cytolytic activity, T-cell co-inhibition, and T-cell co-activation were significantly higher in the low-risk group compared to the high-risk group (**Figure 14L**). CIBERSORT database analysis revealed that the infiltration abundance of M0 macrophages, dendritic cells and monocyte was significantly higher in the high-risk group compared to the low-risk group. Conversely, the infiltration abundance of naive B cells and γδT cells was significantly lower in the high-risk group than in the low-risk group. Furthermore, analyses from multiple immune cell infiltration-related databases demonstrated significant correlations between immune cell infiltration and the risk scores of the prognostic model (**Supplementary Figure S8**). **Supplementary Figure S9** illustrated the correlations between six model genes and the infiltration levels of 22 types of immune cells. The expression of these model genes (with the exception of ITGA7) is predominantly significantly positively correlated with macrophage infiltration, whereas it was significantly negatively correlated with the infiltration of anti-tumor immune cells, such as activated NK cells and CD8+ T cells. In contrast, ITGA7 exhibited an opposite pattern.

### ScRNA-seq analysis of the expression of COL7A1 and its role in PC malignant behaviors

**Figure 15:**
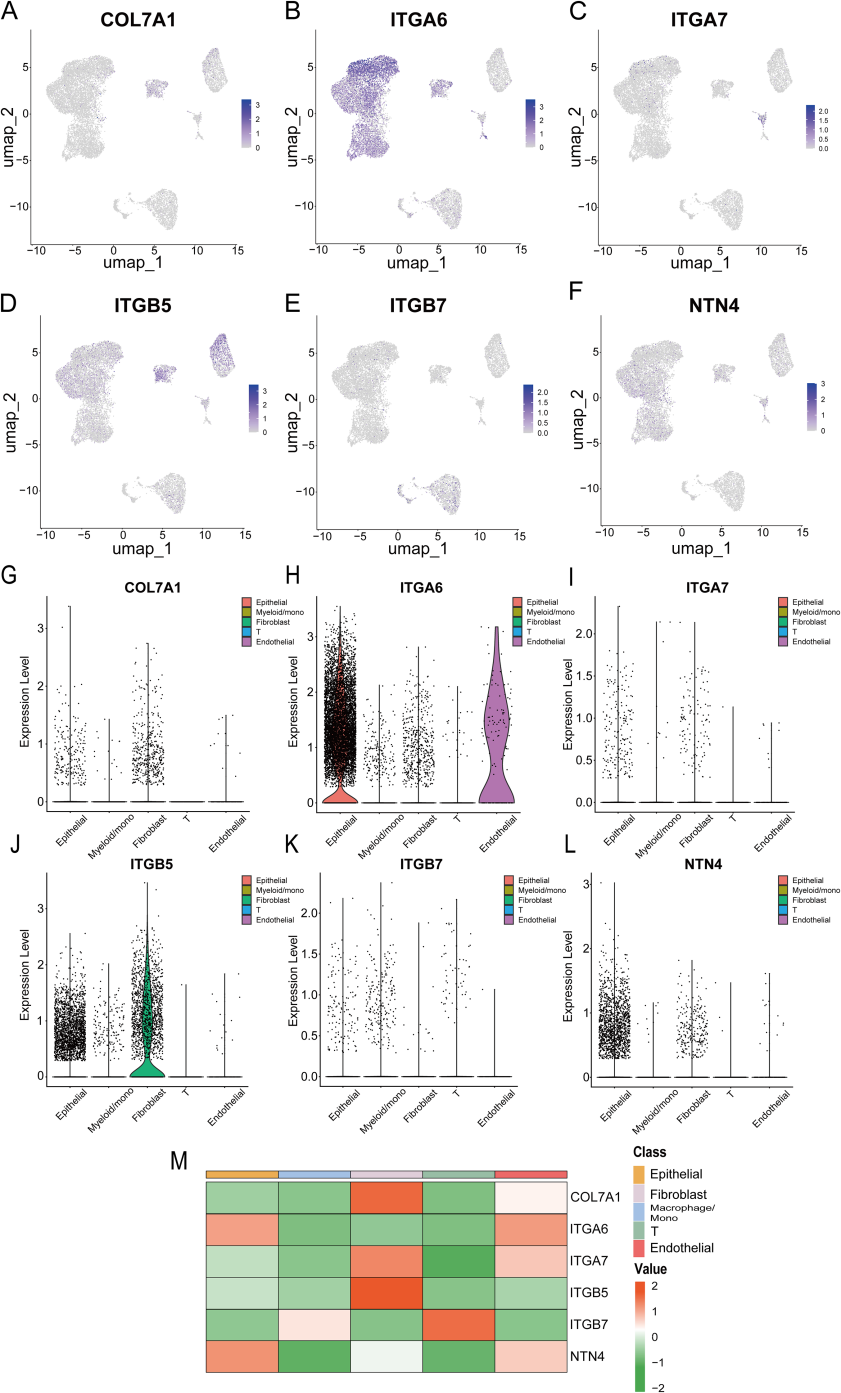
**(A-F)** UMAP plots show the expression of six prognostic model genes in single-cell clusters from the GSE154778 database; **(G-L)** Violin plots of the expression of six prognostic model genes in the GSE154778 database; **(M)** Heatmap of the expression distribution of six prognostic model genes in the GSE154778 database.

Ultimately, we utilized single-cell transcriptome data to analyze the specific expression patterns and distribution of six model genes at the single-cell resolution. In the GSE154778 dataset, analysis demonstrated that COL7A1 and ITGB5 were predominantly expressed in tumor-associated fibroblasts, ITGA6 was detectable in both tumor epithelial and endothelial cells, NTN4 was primarily localized within tumor epithelial cells, whereas ITGA7 and ITGB7 exhibited relatively low expression levels overall (**Figure 15**). The analysis in the GSE197177 dataset showed a high degree of concordance with that in GSE154778 (**Supplementary Figure S10**). In both datasets, the expression patterns and distribution profiles of the six model genes within tumor cells were largely consistent.

Among the six model genes, the potential role of COL7A1 in PC progression has rarely been reported before. To investigate its potential involvement, CAFs were isolated from surgically resected PC tissues and adjacent normal pancreatic tissues. QRT-PCR analysis revealed that COL7A1 expression was significantly upregulated in CAFs compared to normal pancreatic fibroblasts (**Figure 1B**). Both qRT-PCR and western blotting confirmed the knockdown efficiency of three shCOL7A1, among which shCOL7A1–2 exhibited the highest silencing efficiency and was selected for subsequent functional assays (**Figures 1C, D**). Wound healing assays showed that co-culturing PC cells with CAFs enhanced their migratory capacity, whereas COL7A1 knockdown in CAFs largely reversed this effect (**Figure 1E**). Transwell migration assays further indicated that COL7A1 knockdown in CAFs significantly reduced the number of migrated cells (**Figure 1F**). Moreover, when COL7A1 was knocked down in CAFs, the IC50 values of PANC-1 and BxPC-3 cells were significantly lower than those in control groups, indicating increased sensitivity to GEM (**Figures 1G, H**). However, although co-culture with CAFs significantly promoted cell proliferation in colony formation assays, COL7A1 knockdown in CAFs did not significantly affect PC cell proliferation, suggesting that COL7A1 does not play a major role in regulating this process (**Supplementary Figure S11**).

## Discussion

PC remains a highly lethal malignancy at present, therefore, it is crucial to identify effective targets for PC treatment and survival prediction. In this study, we developed a novel prognostic model based on six PCLM and BM-related genes for prognosis prediction, identification of immune microenvironment status, and evaluation of responses to chemotherapy and immunotherapy. We used the TCGA-PAAD cohort as the training cohort to construct the model and utilized the PACA_AU cohort as the validation cohort. The resulting model demonstrated outstanding and robust prognostic performance and accuracy through various methods.Furthermore, enrichment analysis, including GO, KEGG, and GSEA analyses, revealed distinct pathways enriched in the high- and low-risk groups. Moreover, six immune-related algorithms, namely, xCell, CIBERSORT, QUANTISEQ, MCPcounter, EPIC, and ssGSEA, were employed to assess the immune landscape differences between the two risk groups. Subsequently, the immunotherapy responses of the two groups were evaluated using TMB values, TIDE scores, mutation frequencies, and the expression levels of immune checkpoint genes. Additionally, the efficacy of common chemotherapeutics for PC in the two groups was assessed via the OncoPredict R package, and the expression levels of therapeutic targets were explored to evaluate the response to targeted therapy. Finally, the single-cell expression distributions of six prognosis-related genes were analyzed using two single-cell GEO datasets.

In this study, a prognostic model was constructed based on six genes associated with PCLM and BM. These genes have been extensively reported to play critical roles in cancer progression. Notably, four of these genes are integrin-related genes. As transmembrane signaling proteins, integrins are predominantly implicated in promoting tumor progression. ITGA6 was recently reported to be overexpressed in platinum (PT)-resistant epithelial ovarian cancer (EOC), contributing to chemoresistance. Furthermore, ITGA6 facilitates EOC cell dissemination by modulating insulin-like growth factor (IGF) expression and activating the IGF1R and Snail signaling pathways (47). Additionally, multisite m6A modifications of ITGA6 have been identified to promote bladder cancer (BCa) progression. The dCasRx-m6A editor-mediated m6A demethylation of ITGA6 mRNA significantly suppressed BCa cell proliferation and migration both *in vitro* and vivo (48). The role of ITGB7 in tumorigenesis remains controversial. Zhang et al. reported decreased ITGB7 expression in tumor-infiltrating CD8+T cells, with higher expression correlating with improved survival in colorectal cancer patients, suggesting its role in sustaining antitumor immune cell infiltration and inhibiting tumor progression (49). In contrast, in multiple myeloma (MM), oncogenic overexpression of ITGB7 in high-risk cases enhances interactions between malignant plasma B-cells and stromal cells, leading to cell-adhesion-mediated drug resistance (50). In pancreatic cancer (PC), ITGB7 was recently identified as a candidate gene associated with nab-paclitaxel resistance through whole-transcriptome sequencing in PC patient-derived organoids (51). Moreover, ITGB7 has been shown to mediate the oncogenic function of TRIM2, thereby promoting PC progression (52). In our study, we further confirmed ITGB7 expression as a risk factor for poor survival in PC patients, consistent with its established oncogenic role. The involvement of ITGB5 in PC progression has also been recently characterized. Overexpression of N-acetyltransferase 10 (NAT10) was found to enhance perineural invasion (PNI) in PC by stabilizing ITGB5 via N4-acetylcytidine modification, subsequently activating the ITGB5-pFAK-pSrc pathway to promote PNI (53). High ITGB5 expression in PC tissues promotes tumor cell invasion and migration. Additionally, ITGB5 contributes to DNA damage repair and activates the MEK/ERK signaling pathway, thereby conferring intrinsic radiation resistance (54). ITGA7 exhibits context-dependent expression patterns across various cancers. Traditionally recognized as a tumor suppressor, ITGA7 is significantly downregulated in breast cancer stem cells (BCSCs)-key contributors to therapy resistance and adverse clinical outcomes. Low ITGA7 expression correlates with reduced survival in chemotherapy-treated patients, highlighting its potential as a predictive biomarker for treatment response (55). Promoter hypermethylation has been shown to suppress ITGA7 expression, leading to activation of the PI3K/AKT/NF-κB pathway and enhanced proliferation and migration in colorectal cancer (56). NTN4, an epigenetically regulated gene, plays a dual role in cancer metastasis. In clear cell renal cell carcinoma (ccRCC), NTN4 inhibits tumor progression by regulating β-catenin expression and nuclear translocation (57). Conversely, in endometrial cancer, NTN4 exhibits oncogenic properties. EXOSC5 upregulates NTN4 expression, activating c-MYC through the integrin β1/FAK/SRC pathway to sustain cancer stem cell activity (58). Current evidence on COL7A1 in cancer progression remains limited and largely derived from bioinformatic studies. As a BM-related prognostic marker, COL7A1 demonstrated strong prognostic performance and immune microenvironment predictive capacity in lung cancer (59). It effectively stratified prognosis in ccRCC and showed robust prognostic value (60). Ding et al. reported elevated COL7A1 expression in PC, which is associated with patient survival and specific immune cell infiltration (61). In our study, COL7A1 expression in CAFs was linked to enhanced PC cell migration but did not significantly influence cell proliferation. This observation aligns closely with our prior bioinformatic findings, supporting COL7A1 as a potential therapeutic target for suppressing PC metastasis.

The efficacy of immunotherapy is heavily contingent upon the immune landscape of cancer. PC displays an immunologically “cold” tumor microenvironment (TME), marked by significant myeloid cells infiltration, a paucity of CD8+T cells, and low expression of activation markers. These characteristics are indicative of absent or dysfunctional adaptive T-cell immunity and contribute to resistance against immune checkpoint blockade (ICB) (62). Moreover, in addition to its classical oncogenic role, accumulating evidence highlights that mutant KRAS plays a critical role in establishing an immunosuppressive TME, which underpins PC’s resistance to immunotherapy (63). Recently, a study revealed that combining a KRAS inhibitor with immunotherapy agents *in vivo* not only enhanced T-cell infiltration and activation but also depleted immunosuppressive myeloid cells and alleviated the immunosuppressive TME in PC, thereby extending the survival of an autochthonous PC mouse model (64). Given the limited efficacy of immunotherapy in PC, predicting the response to immunotherapy could facilitate the identification of specific patients who are more likely to benefit from this treatment. In our study, we developed a novel risk prediction model to evaluate the response to immunotherapy. Using this model, we observed that the KRAS mutation rate was significantly higher in the high-risk group compared to the low-risk group. Additionally, the TIDE scores were markedly elevated in the high-risk group, suggesting a poorer response to immunotherapy. Regarding the underlying mechanism, CD8+ T-cell infiltration was substantially higher in low-risk PC tissues than in high-risk PC tissues, potentially contributing to immunotherapy resistance in the high-risk group. These findings indicate that our model can effectively identify PC patients who are more likely to benefit from immunotherapy, thereby enhancing therapeutic outcomes. Numerous recent studies have underscored the synergistic therapeutic effects of combining targeted therapy with immunotherapy. TMOD3 was highly expressed in PC tissues, modulating immunotherapy resistance. A TMOD3 inhibitor demonstrated a synergistic effect with PD-1 antibody in PC treatment (65). Furthermore, high OSBPL3 expression indicated an immunosuppressive microenvironment characterized by reduced CD8+T cell infiltration and increased Treg cells and M2 macrophages, which might serve as a promising therapeutic target (66). However, whether the genes in our model can function as targets to sensitize immunotherapy remains to be further explored.

In the present study, a novel PCLM- and BM-related model was developed to predict prognosis, immune microenvironment status, and responses to immunotherapy and chemotherapy, with the aim of identifying new therapeutic targets for PC. Although this model demonstrates improved performance and accuracy compared to previous models, several limitations still remain. First, our findings primarily rely on data from public databases, which lack real-world clinical validation to confirm the model’s accuracy and applicability. Second, the potential for batch effects between datasets caused a less satisfactory model evaluation effect. This discrepancy might be attributed to sample heterogeneity, treatment differences and the relatively small size of training cohort. Therefore, larger and more diverse cohorts will be needed for further model validation. Third, although we conducted preliminary cell experiments to support the bioinformatic analysis, the functional roles and underlying mechanisms of certain biomarkers identified in this study, such as COL7A1, have not been fully validated through *in vitro* functional assays or *in vivo* animal models. Therefore, further experimental studies are warranted to address these limitations.

## Conclusion

In conclusion, a model based on six PCLM and BM-related genes was developed to effectively predict prognosis, immune microenvironment status, and response to immunotherapy. Therefore, our findings offer promising insights that could assist physicians in making more accurate and personalized treatment decisions for patients with PC.

## Data Availability

The original contributions presented in the study are included in the article/**Supplementary Material**. Further inquiries can be directed to the corresponding author.

## Supplementary material

Supplementary Figure 1
**(A)** The original tSNE plot of GSE197177; **(B)** The original UMAP plot of GSE197177; **(C)** The tSNE plot of GSE197177 after simple annotation by the SingleR R package; **(D)** The UMAP plot of GSE197177 after simple annotation by the SingleR R package; **(E)** The tSNE plot of GSE197177 after manual marker annotation; **(F)** The UMAP plot of GSE197177 after manual marker annotation; **(G)** Dot plot of expression levels of each cell population in manual annotation; **(H)** Violin plot of expression levels of each cell population in manual annotation; **(I)** Volcano plot of GSE154778, showing significantly upregulated and downregulated differentially expressed genes (DEGs).

Supplementary Figure 2
**(A)** Heatmap of the top 50 upregulated and downregulated DEGs between primary tumors and metastases in GSE71729; **(B)** Volcano plot of DEGs between primary tumors and metastases in GSE71729; **(C)** Selecting the optimal β parameter for WGCNA analysis in GSE71729; **(D)** System tree diagram of gene set clusters from WGCNA analysis; **(E)** Module diagram of co-expressed genes from WGCNA analysis.

Supplementary Figure 3 The expression levels of 6 genes in high-risk and low-risk pancreatic cancer patient groups.

Supplementary Figure 4 Kaplan-Meier survival curves of PCLM and BM-related six model genes in pancreatic cancer patients.

Supplementary Figure 5
**(A)** Correlation analysis of tumor grade and risk score in the TCGA cohort; **(B)** Correlation analysis of tumor T stage and risk score in the TCGA cohort; **(C)** Correlation analysis of tumor N stage and risk score in the TCGA cohort; **(D)** Correlation analysis of tumor M stage and risk score in the TCGA cohort; **(E)** Correlation analysis of tumor stage and risk score in the TCGA cohort; **(F)** Correlation analysis of tumor grade and risk score in the ICGC cohort; **(G)** Correlation analysis of tumor T stage and risk score in the ICGC cohort; **(H)** Correlation analysis of tumor N stage and risk score in the ICGC cohort; **(I)** Correlation analysis of tumor M stage and risk score in the ICGC cohort; **(J)** Correlation analysis of tumor stage and risk score in the ICGC cohort.

Supplementary Figure 6
**(A)** Univariate analysis of the ICGC cohort; **(B)** Multivariate analysis of the ICGC cohort; **(C)** Univariate analysis of the ICGC cohort, including the nomogram model; **(D)** Multivariate analysis of the ICGC cohortt, including the nomogram model; **(E)** The 1-, 2-, and 3-year survival prediction accuracy of the ROC curve prediction model of the ICGC dataset; **(F)** Multivariate ROC curves were drawn for gender, age, stage, grade, risk score of the prediction model, and the prognostic prediction efficacy of the nomogram; **(G)** Calibration curves for 1-, 2-, and 3-year survival were used to evaluate the prognostic prediction stability of the nomogram; **(H)** DCA curves demonstrated the clinical benefit levels of the nomogram model at 1-, 2-, and 3-years; **(I)** DCA curves demonstrated the corresponding clinical benefits of the nomogram model, risk score of the model, and other clinical variables; **(J)** C-index curves were used to evaluate the prognostic efficacy of the nomogram, risk score of the prognostic model, and related clinical variables.

Supplementary Figure 7 Correlation diagram of six PCLM and BM-related prognostic model genes with tumor marker signaling pathways.

Supplementary Figure 8
**(A-C)** CIBERSORT was used to analyze the differences in immune cell infiltration between high-risk and low-risk groups in the TCGA database; **(D)** The correlations between immune cell infiltration levels and risk scores in the TCGA database were analyzed using XCELL, CIBERSORT, QUANTISEQ, MCPCOUNTER, EPIC and CIBERSORT-ABS. * p<0.05; ** p<0.01; *** p<0.001.

Supplementary Figure 9 The lollipop plot shows the correlation analysis between six model genes and immune cell infiltration.

Supplementary Figure 10
**(A-F)** UMAP plots show the expression of six prognostic model genes in single-cell clusters from the GSE197177 database; **(G-L)** Violin plots of the expression of six prognostic model genes in the GSE197177 database; **(M)** Heatmap of the expression distribution of six prognostic model genes in the GSE197177 database.

Supplementary Figure 11 The results of colony formation assay and statistical analysis. ** p<0.01.
