# Supplementary figures and images for: Integration of bulk and single-cell transcriptomic data reveals a novel signature related to liver metastasis and basement membrane in pancreatic cancer
Source: Front Immunol. 2025 Oct 29;16:1671956. doi: 10.3389/fimmu.2025.1671956 (PMC12605406; doi:10.3389/fimmu.2025.1671956)

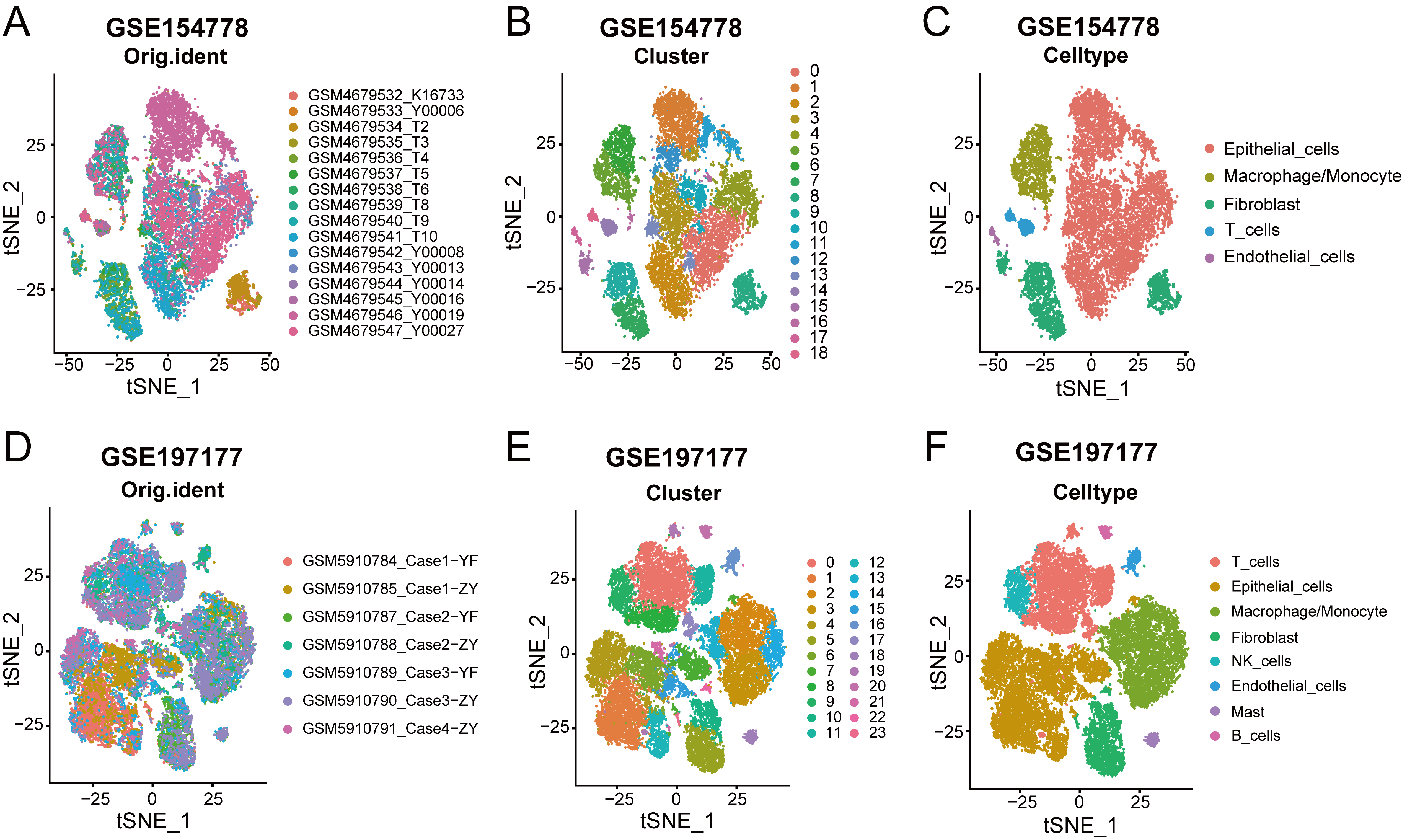

Supplement: Supplementary Figure 1 — (A) The original tSNE plot of GSE197177; (B) The original UMAP plot of GSE197177; (C) The tSNE plot of GSE197177 after simple annotation by the SingleR R package; (D) The UMAP plot of GSE197177 after simple annotation by the SingleR R package; (E) The tSNE plot of GSE197177 after manual marker annotation; (F) The UMAP plot of GSE197177 after manual marker annotation; (G) Dot plot of expression levels of each cell population in manual annotation; (H) Violin plot of expression levels of each cell population in manual annotation; (I) Volcano plot of GSE154778, showing significantly upregulated and downregulated differentially expressed genes (DEGs). [file Image1.tif]

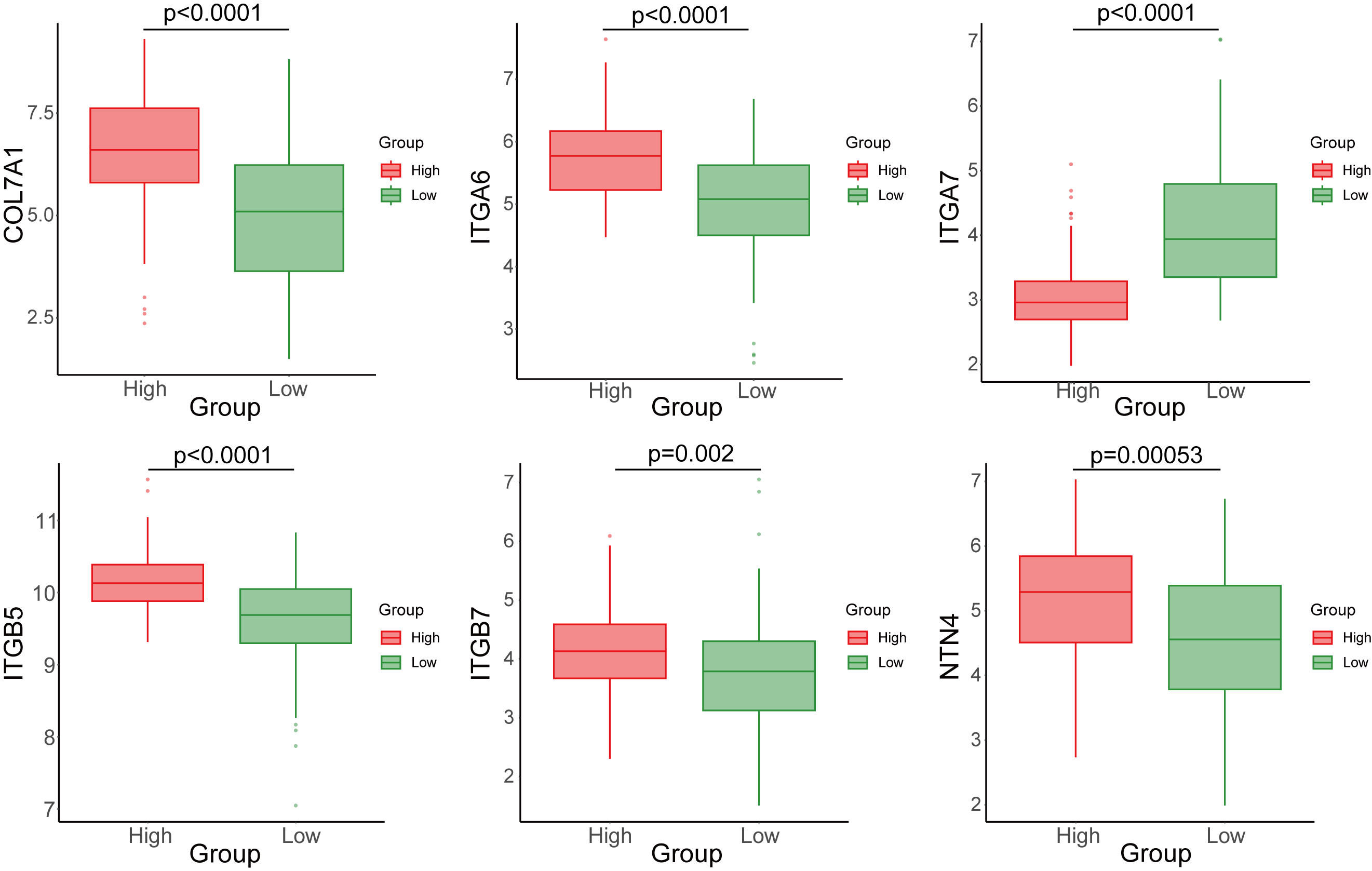

Supplement: Supplementary Figure 3 — The expression levels of 6 genes in high-risk and low-risk pancreatic cancer patient groups. [file Image3.tif]

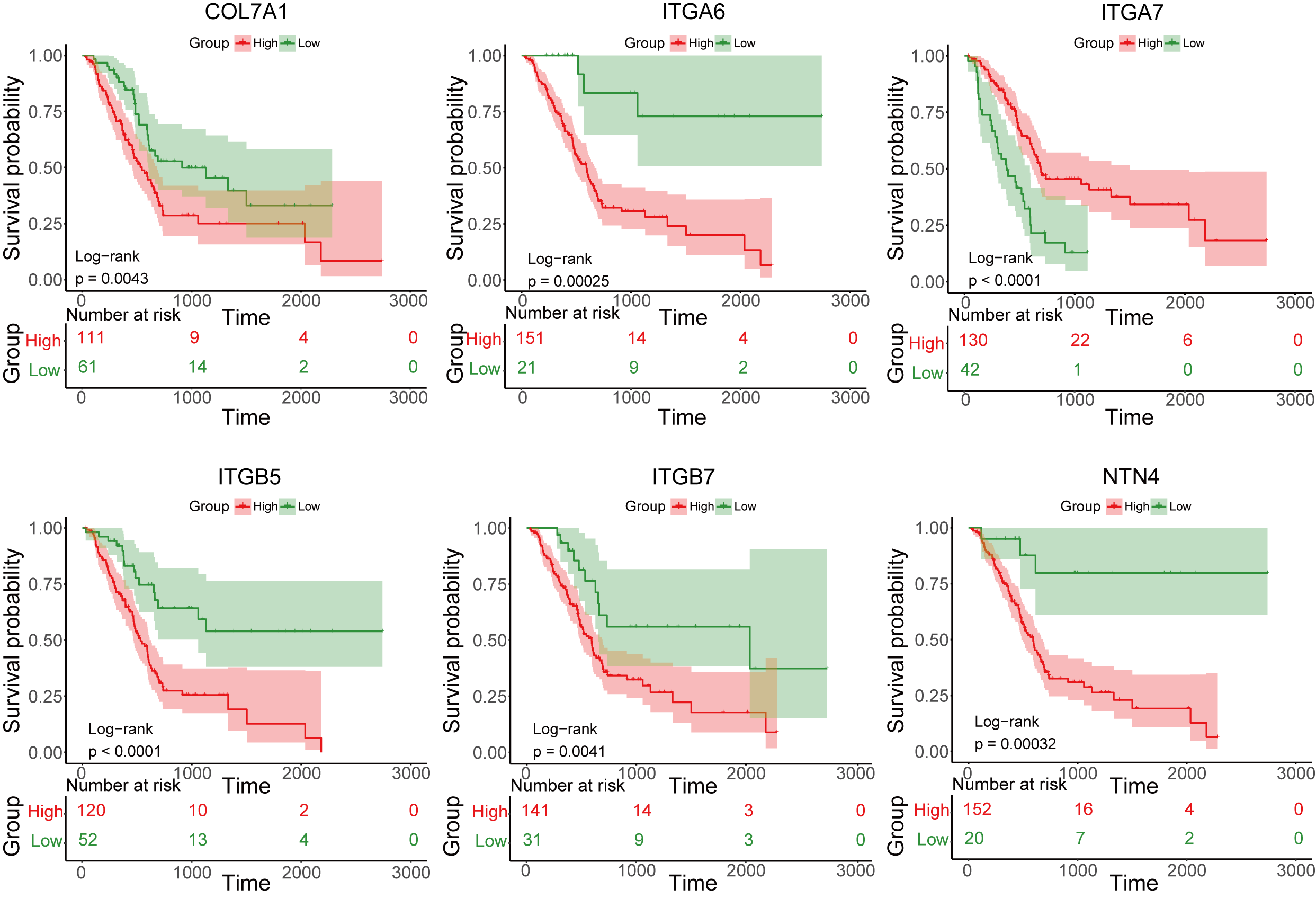

Supplement: Supplementary Figure 4 — Kaplan-Meier survival curves of PCLM and BM-related six model genes in pancreatic cancer patients. [file Image4.tif]

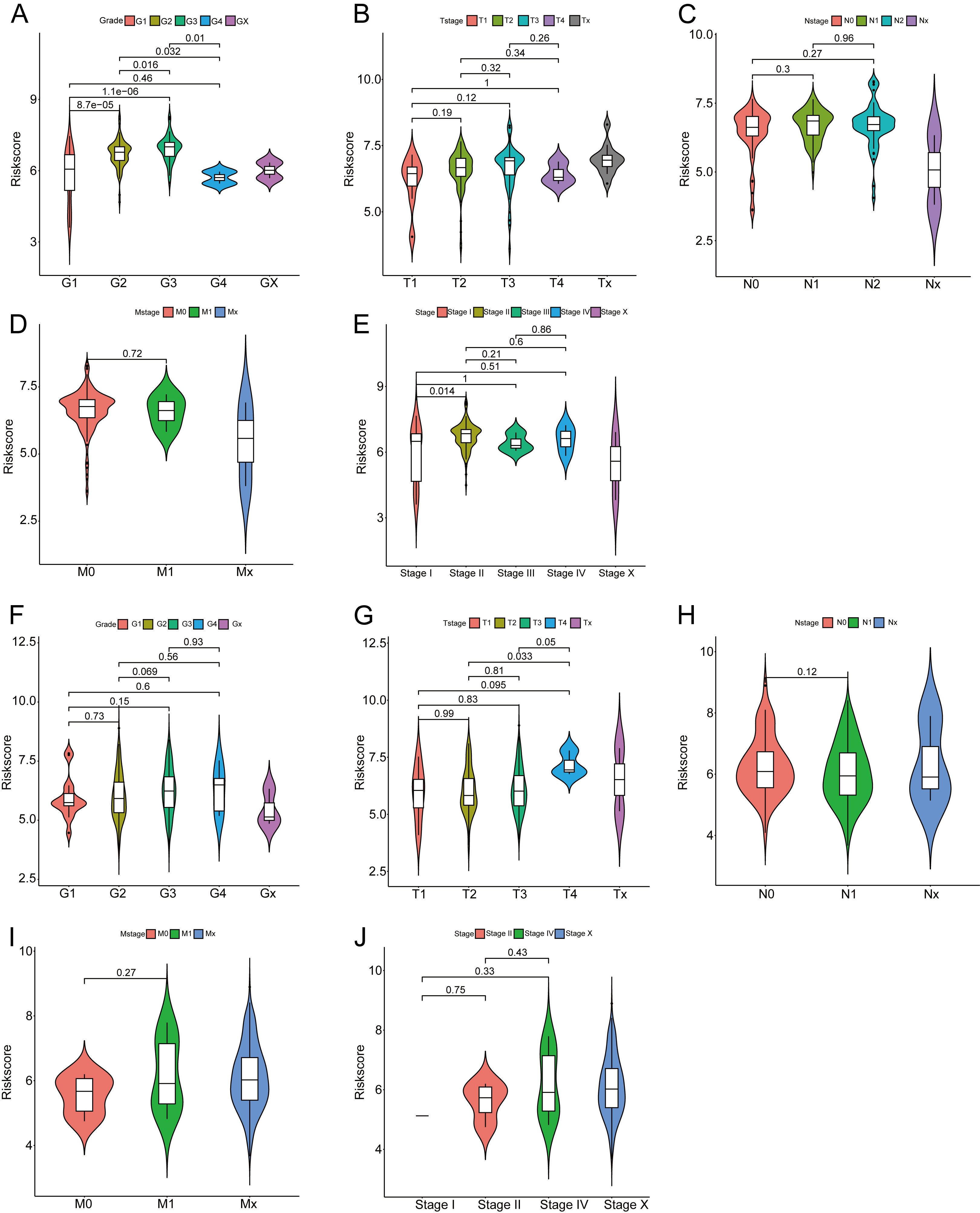

Supplement: Supplementary Figure 5 — (A) Correlation analysis of tumor grade and risk score in the TCGA cohort; (B) Correlation analysis of tumor T stage and risk score in the TCGA cohort; (C) Correlation analysis of tumor N stage and risk score in the TCGA cohort; (D) Correlation analysis of tumor M stage and risk score in the TCGA cohort; (E) Correlation analysis of tumor stage and risk score in the TCGA cohort; (F) Correlation analysis of tumor grade and risk score in the ICGC cohort; (G) Correlation analysis of tumor T stage and risk score in the ICGC cohort; (H) Correlation analysis of tumor N stage and risk score in the ICGC cohort; (I) Correlation analysis of tumor M stage and risk score in the ICGC cohort; (J) Correlation analysis of tumor stage and risk score in the ICGC cohort. [file Image5.tif]

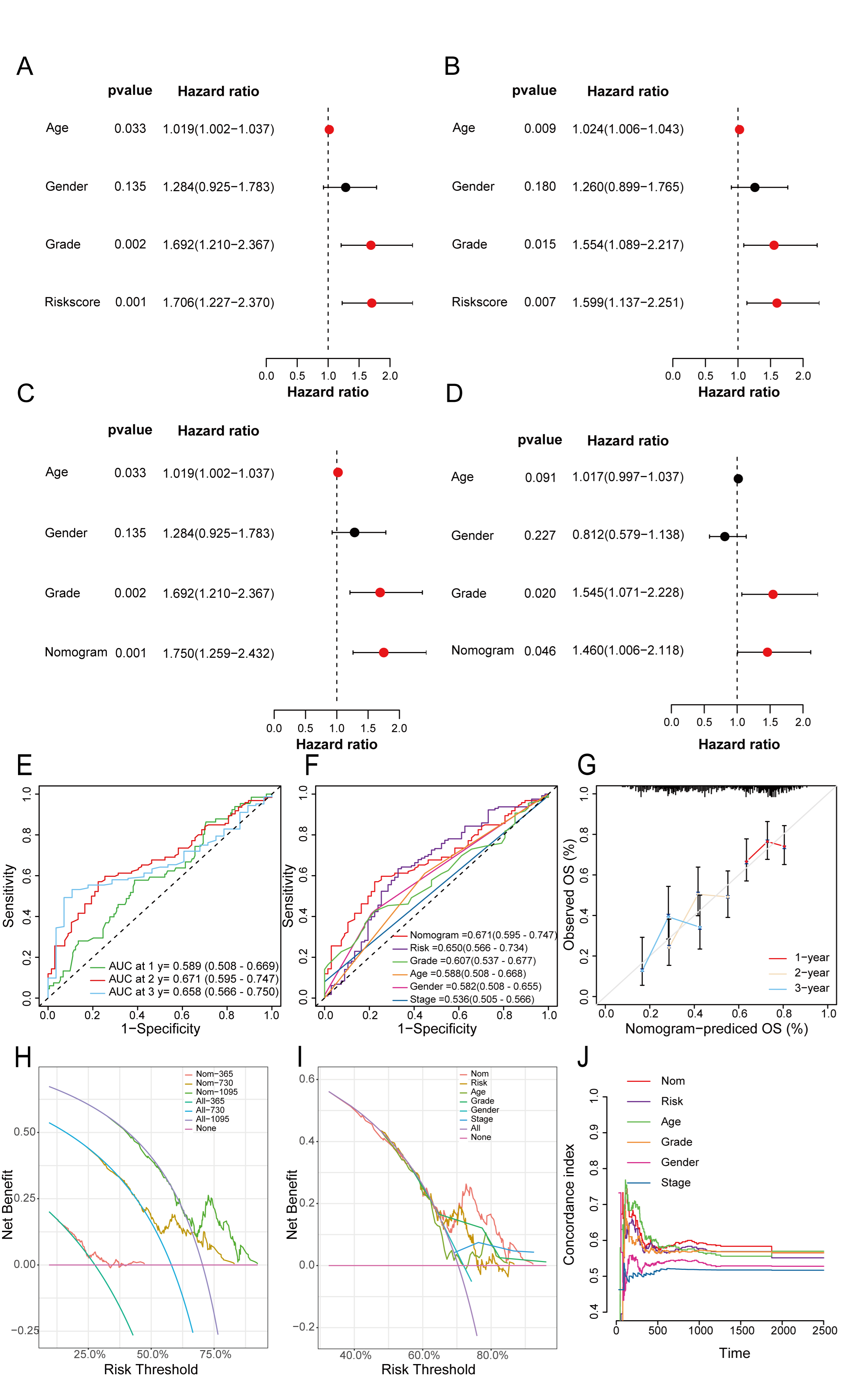

Supplement: Supplementary Figure 6 — (A) Univariate analysis of the ICGC cohort; (B) Multivariate analysis of the ICGC cohort; (C) Univariate analysis of the ICGC cohort, including the nomogram model; (D) Multivariate analysis of the ICGC cohortt, including the nomogram model; (E) The 1-, 2-, and 3-year survival prediction accuracy of the ROC curve prediction model of the ICGC dataset; (F) Multivariate ROC curves were drawn for gender, age, stage, grade, risk score of the prediction model, and the prognostic prediction efficacy of the nomogram; (G) Calibration curves for 1-, 2-, and 3-year survival were used to evaluate the prognostic prediction stability of the nomogram; (H) DCA curves demonstrated the clinical benefit levels of the nomogram model at 1-, 2-, and 3-years; (I) DCA curves demonstrated the corresponding clinical benefits of the nomogram model, risk score of the model, and other clinical variables; (J) C-index curves were used to evaluate the prognostic efficacy of the nomogram, risk score of the prognostic model, and related clinical variables. [file Image6.png]

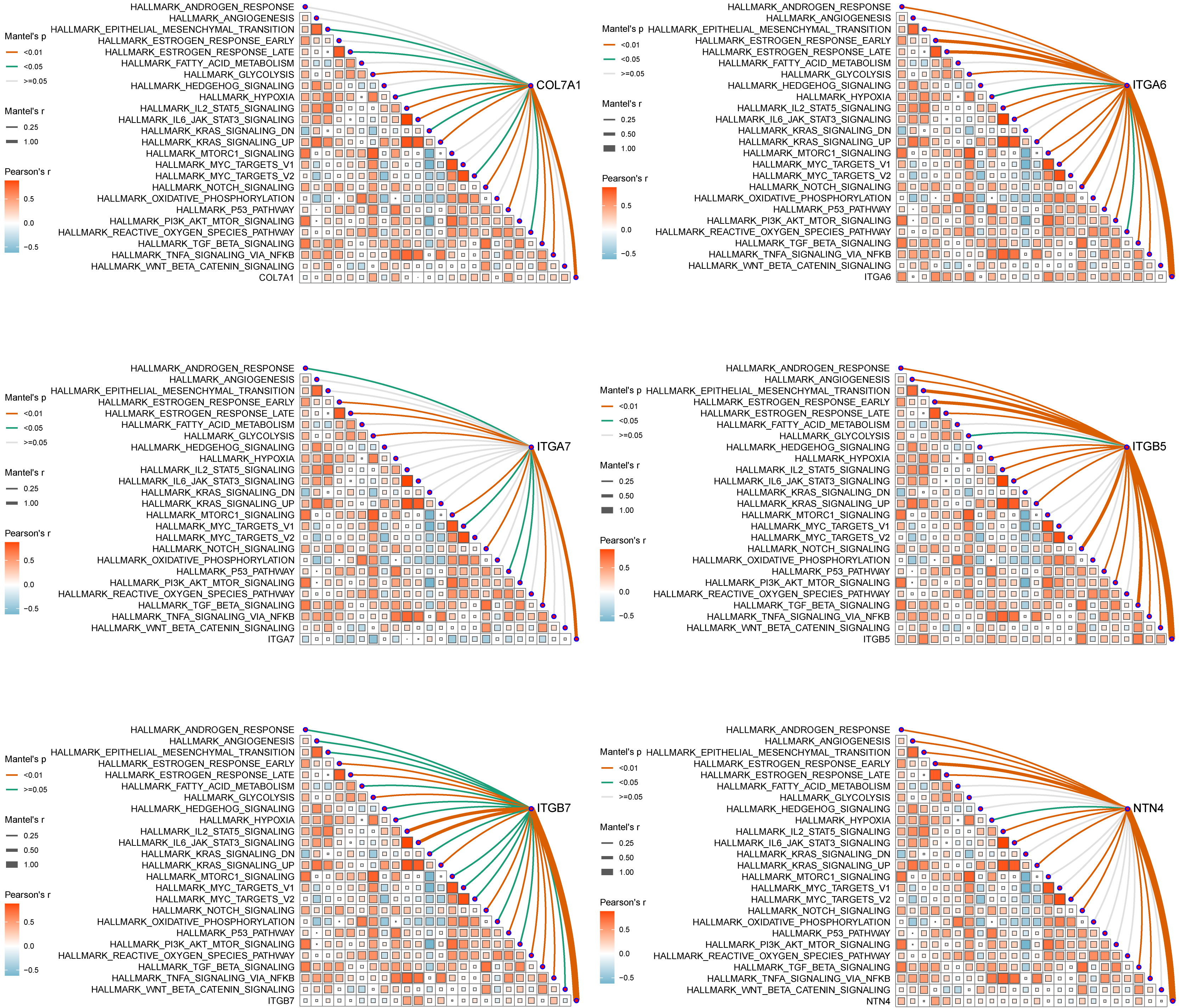

Supplement: Supplementary Figure 7 — Correlation diagram of six PCLM and BM-related prognostic model genes with tumor marker signaling pathways. [file Image7.tif]

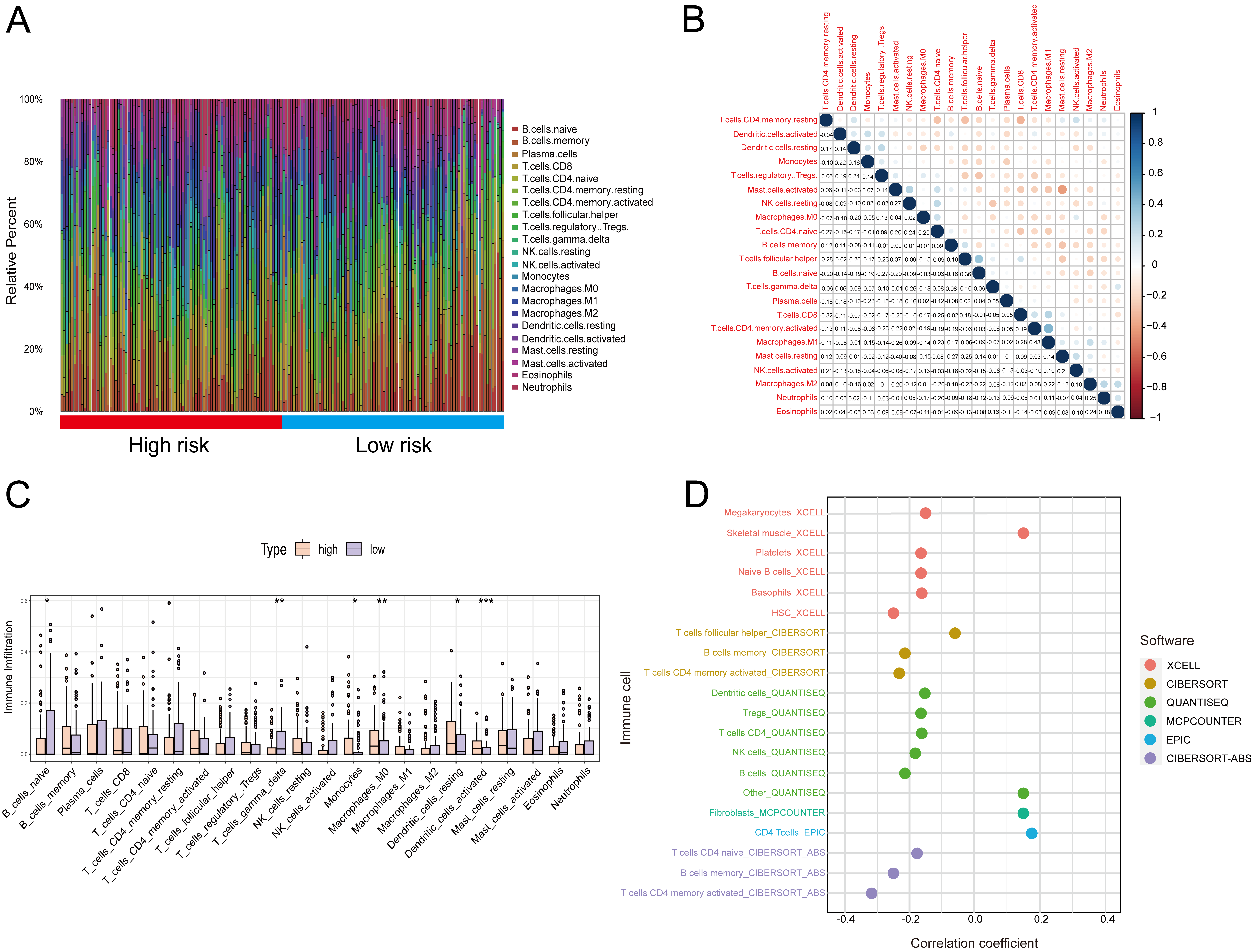

Supplement: Supplementary Figure 8 — (A-C) CIBERSORT was used to analyze the differences in immune cell infiltration between high-risk and low-risk groups in the TCGA database; (D) The correlations between immune cell infiltration levels and risk scores in the TCGA database were analyzed using XCELL, CIBERSORT, QUANTISEQ, MCPCOUNTER, EPIC and CIBERSORT-ABS. * p<0.05; ** p<0.01; *** p<0.001. [file Image8.tif]

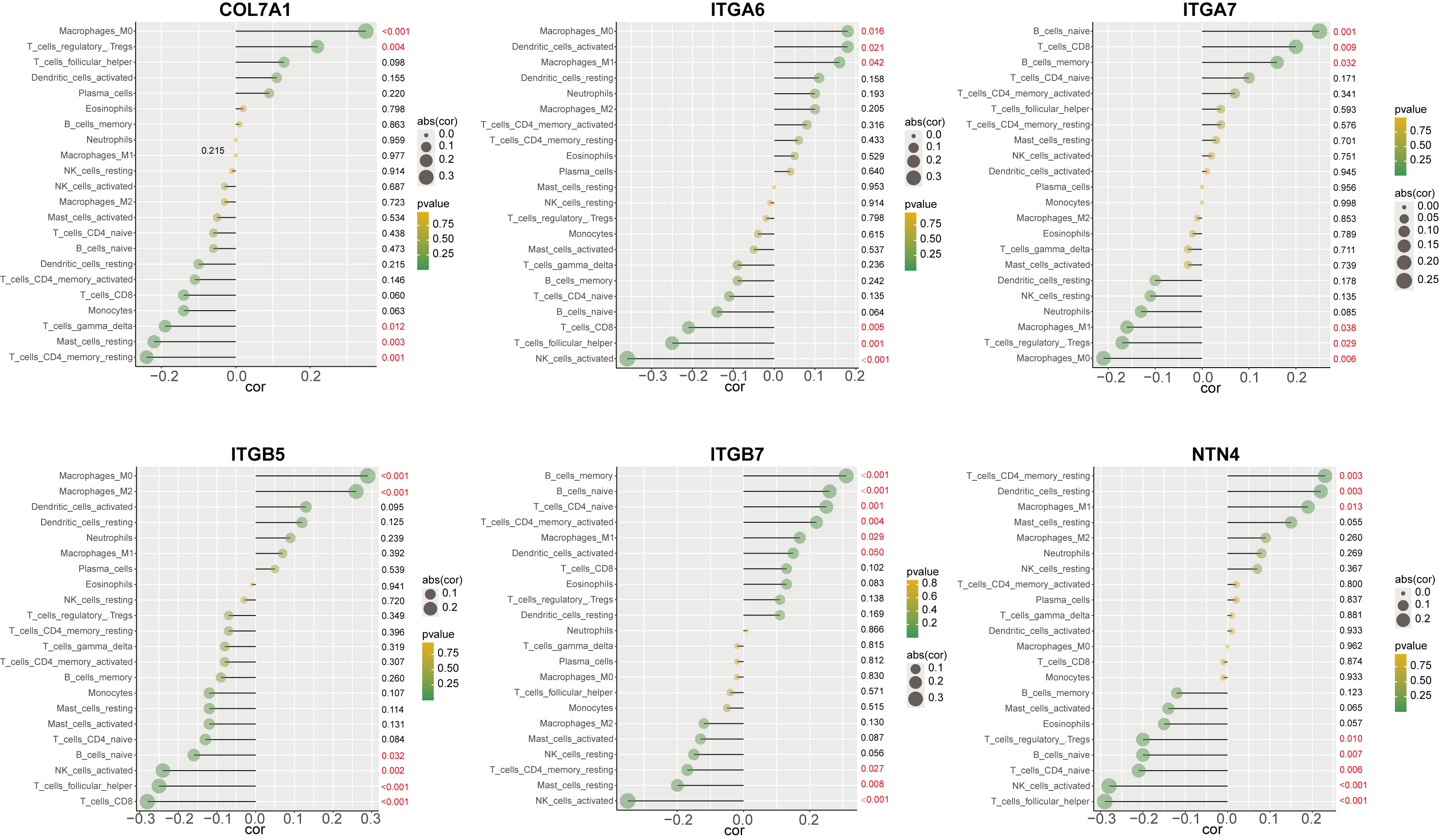

Supplement: Supplementary Figure 9 — The lollipop plot shows the correlation analysis between six model genes and immune cell infiltration. [file Image9.tif]

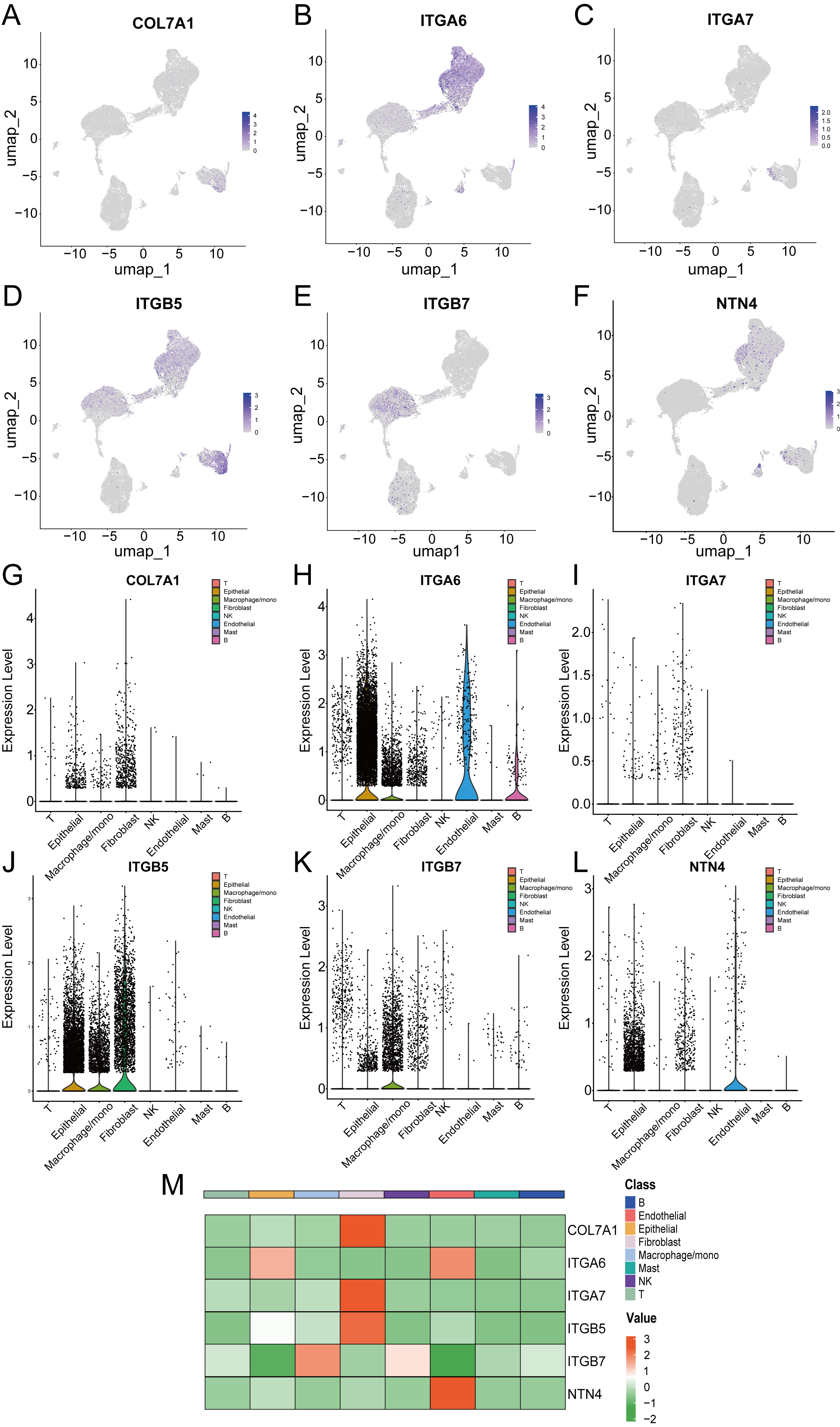

Supplement: Supplementary Figure 10 — (A-F) UMAP plots show the expression of six prognostic model genes in single-cell clusters from the GSE197177 database; (G-L) Violin plots of the expression of six prognostic model genes in the GSE197177 database; (M) Heatmap of the expression distribution of six prognostic model genes in the GSE197177 database. [file Image10.tif]

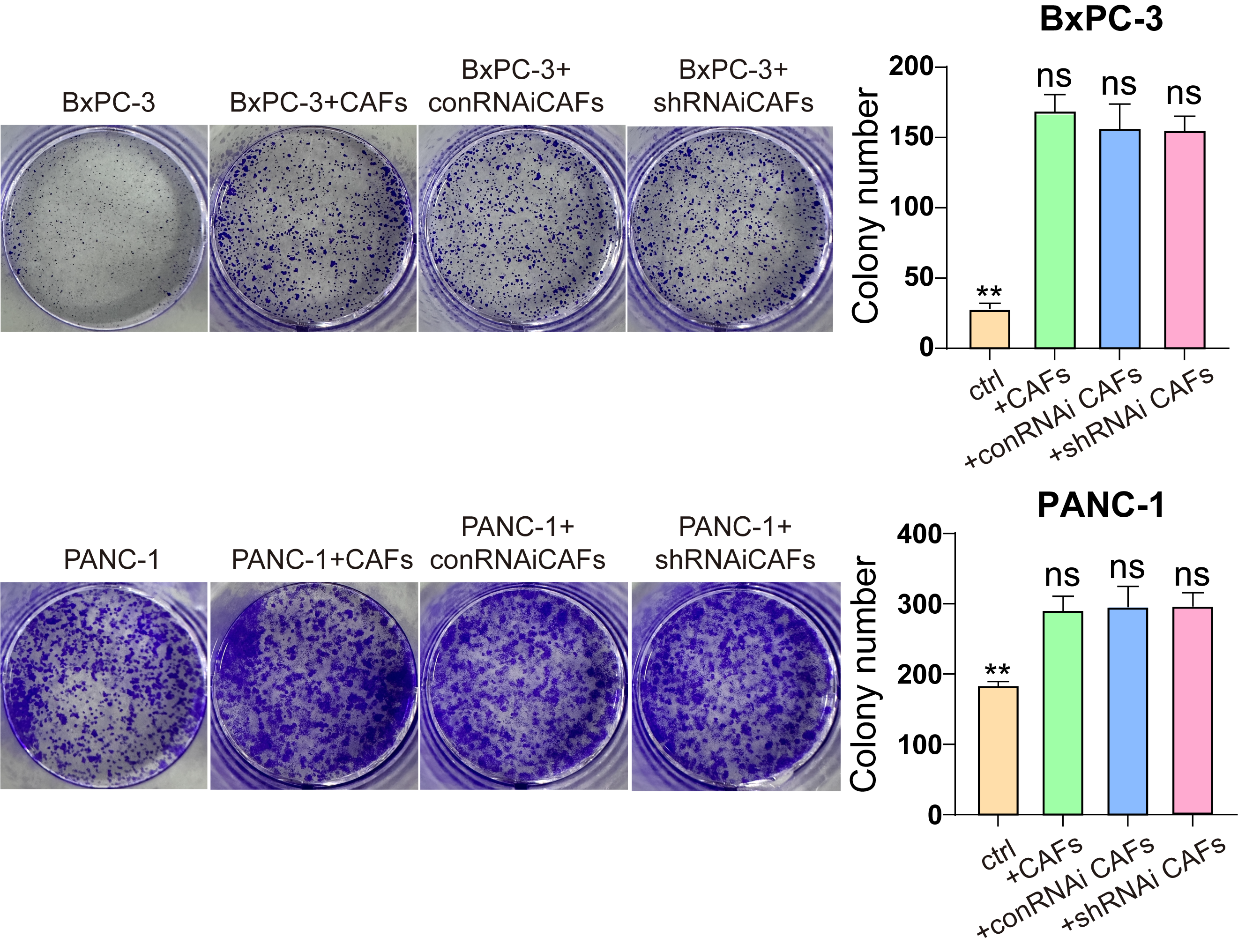

Supplement: Supplementary Figure 11 — The results of colony formation assay and statistical analysis. ** p<0.01. [file Image11.tif]
