# Supplementary material for: Integration of bulk and single-cell transcriptomic data reveals a novel signature related to liver metastasis and basement membrane in pancreatic cancer
Source: Front Immunol. 2025 Oct 29;16:1671956. doi: 10.3389/fimmu.2025.1671956 (PMC12605406; doi:10.3389/fimmu.2025.1671956)
Supplement: Supplementary file 12 [file DataSheet1.docx]

Supplementary Material

**Zooplankton and micronekton active flux around the Iberian Peninsula**

**María Couret^1*^, Airam N. Sarmiento-Lezcano^1,2^, José María Landeira^3^, Sarah L.C. Giering^4^, Will Major^4^, M. Pilar Olivar^5^, Javier Díaz-Pérez^1^, Arturo Castellón^6^, Santiago Hernández-León^1^.**

^1^ Instituto de Oceanografía y Cambio Global, IOCAG, Universidad de Las Palmas de Gran Canaria, Unidad Asociada ULPGC-CSIC, Campus de Taliarte, 35214 Telde, Gran Canaria, Canary Islands, Spain.

^2^ Centro Oceanográfico de A Coruña, Instituto Español de Oceanografía, IEO, 15080 A Coruña, Spain.

^3^ Department of Biology, Norwegian University of Science and Technology, Trondhjem Biological Station NO-7491 Trondheim, Norway

^4^ Ocean BioGeosciences, National Oceanography Centre, Southampton, European Way, Southampton, UK.

^5^ Institut de Ciències del Mar (CSIC). Passeig Marítim 37-49, Barcelona 08003, Spain.

^6^ Unidad de Tecnología Marina (CSIC). Passeig Marítim 37-49, Barcelona 08003, Spain.

*** Correspondence:**María Couret
[maria.couret@ulpgc.es](mailto:maria.couret@ulpgc.es) [mariacouhuertas@gmail.com](mailto:mariacouhuertas@gmail.com)

# Supplementary Material

**SM1. Study area.**

The south of the Mallorca channel (Balearic Islands, western Mediterranean, Z1) is characterized by the oligotrophy of its waters but considered as a hotspot of biological activity and biodiversity in the deep-sea (Massutí et al., 2021). In contrast, the Alborán Sea (Z2), unlike the general oligotrophy dominating most of the Mediterranean, holds two anticyclonic gyres of high biological productivity (Bárcena et al., 2004). This area together with the Gulf of Cádiz (Z3) is considered a transition zone between the Atlantic Ocean and the Mediterranean Sea, with water-mass exchange through the Strait of Gibraltar. This region couples the convergence of critical water masses regarding the Atlantic Meridional Overturning Circulation with a semi-permanent upwelling regime, itself connected to the larger dynamic cells off northwest Africa (Penaud et al., 2016). The area off Lisbon (Portugal, Z4) is characterized by a coastal upwelling, intensified during the summer when upwelled waters occupy the surface layers over the whole western shelf and part of the upper slope off Portugal (Fiuza, 1983). This scenario is also found in the Galician coast (Z5), showing upwelling favorable conditions during spring-summer (Villegas-Ríos et al., 2011).

**SM2. Electron transfer system activity.**

Frozen samples were homogenized at the laboratory with a Teflon pestle in Tris-EDTA buffer at 0-4°C, to avoid degradation of enzyme activity and proteins. Then, the homogenates were centrifuged at 4000 rpm at 0°C for 10 min. An aliquot was subsampled from the homogenate and incubated, at 16°C for zooplankton and 18°C for micronekton, at darkness using NADH, NADPH, succinate, and a tetrazolium salt (INT) as the artificial electron acceptor. After 20 min, the incubation was stopped with a quench solution. The ETS activity was estimated spectrophotometrically at 490 nm with a turbidity baseline of 750 nm. In order to correct ETS activity for in situ temperature (200-700 m layer, considered as the residence depth of migrants, Hays, 2003), we used the Arrhenius equation and an activation energy of 15 kcal⋅mol^-1^ (King and Packard, 1975). Protein content was determined using the method of Lowry et al. (1951) modified by Rutter (1967), and using bovine serum albumin (BSA) as the standard. Zooplankton protein content was converted to dry weight (DW) using the ratio of 2.49 ± 1.73 recently given by Hernández-León et al. (2019c) for zooplankton in subtropical waters. ETS activity in micronekton was measured in the whole animal. Micronekton protein content was converted to DW using the ratio given by Bailey et al. (1995) of 2.21 ± 0.45 for mesopelagic fish, and 2.48 ± 1.09 for decapods. We calculated ETS as the mean of all zooplankton and micronekton groups to estimate the overall community-level metabolic rate.

**SM3. Active flux**.

Zooplankton and micronekton active fluxes (AF) were estimated as the sum of the respiratory (RF), mortality (MF), gut flux (GF), and excretion (EF):

AF = RF (Eq. S1-S3) + MF (Eq. S4ab) + GF (Eq. S5ab) + EF (Eq. S6) (Eq. 1)

First, respiration at depth (R_depth_) was determined using the average ETS activity and a quite conservative respiration to ETS (R/ETS) ratio of 0.5 ± 0.1, according to values ranging from 0.46 to 0.96 for zooplankton >1 mm (Hernández-León and Gómez, 1996), migrant copepods (Hernández-León et al., 2019c), euphausiids (Couret et al., 2024), and fish (Ikeda, 1989) (Eq. S1).

R_depth_ = $\int_{200}^{700} ETS$ × (0.5 ± 0.1) (Eq. S1)

Respiration (R) was converted into carbon units using a respiratory quotient (CO_2_ respired/O_2_ consumed) of 0.97 ± 0.4 (Omori and Ikeda, 1984) (Eq. S2).

R = R_depth_  × (0.97 ± 0.4) (Eq. S2)

RF was then estimated multiplying respiration by the migrant biomass (MB), assuming a mesopelagic residence time of 12 h (Ariza et al., 2015) (Eq. S3)

RF = R × (MB × 0.5) (Eq. S3)

Zooplankton mortality flux (MF*zooplankton*) was estimated assuming steady-state conditions in the mesopelagic zone (growth= mortality), using the equation of Ikeda and Motoda (1978) that relates respiration and growth applying assimilation efficiencies of 30 and 70%, respectively (see review in Omori and Ikeda, 1984) (Eq. S4a).

MF_zooplankton_ = RF × (0.75 ± 0.19) (Eq. S4a)

For micronekton, mortality flux (MF_micronekton_) was estimated from growth assuming steady state conditions and using the growth/metabolism ratio of 0.66 ± 0.17 (Brett and Groves, 1979) (Eq. S4b).

MF_micronekton_= RF × (0.66 ± 0.17) (Eq. S4b)

Zooplankton gut flux (GF_zooplankton_) was estimated assuming that feeding is 2.5 ± 0.5 times R (Ikeda and Motoda, 1978), and that migrant zooplankton egested 50% of the gut content at depth (Ariza et al., 2015) (Eq. S5a).

GF_zooplankton_ = RF × (2.5 ± 0.5 × 0.5) (Eq. S5a)

For micronekton gut flux (GF_micronekton_), we assumed that carnivorous organisms egest an amount equivalent to the 40% of the respired carbon (Brett and Groves, 1979), and they transport feces to the mesopelagic because of their density and the long gut passage time of large animals. Assuming that micronektonic migrants egest after the downward migration, the egestion should be double in relation to respiration during 24 h. Therefore, we used an egestion equivalent to 80 ± 16% of the respired carbon (Ariza et al., 2015) (Eq. S5b).

GF_micronekton_ = RF × (0.8 ± 0.16) (Eq. S5b)

EF was estimated using the values by Steinberg et al. (2000), who reported that excretion makes up 24 ± 26% of the respired plus excreted carbon (Eq. S6).

EF = RF × (0.24 ± 0.26) (Eq. S6)

**SM4. Sensitivity analysis.**

To account for the uncertainties and potential error propagation, we carried out a sensitive analysis for the biomass and metabolic rate estimates of both zooplankton using the Monte Carlo method with a range of conversion factors (CF). Zooplankton carbon weight (CW_zooplankton_) was estimated by converting the image area (in pixels) to DW (a_DW_ and b_DW_) and then to carbon (CF_CW_) (Eq. 2).

CW_zooplankton_ = (*a*_DW_ ⨉ Area ^ *b*_DW_ ) ± δ_DW_ ⨉ CF_CW_ ± δ_CW_ (Eq. 2)

For the conversion of zooplankton area (in pixels) to DW (a_DW_ and b_DW_), we used the equations provided in Table 1 by Lehette and Hernández-León (2009). We used the organism-specific regressions when possible (ostracods, chaetognaths, slaps, siphonophores, subtropical euphausiids, subtropical copepods, for which the number of observations was, respectively, n = 3551, 1678, 682, 159, 637, 52396). For the remaining organisms, we used the regression for general mesozooplankton (n = 6115). We assumed the error on these estimates based on the standard error of the regression slope for general mesozooplankton (slope = 1.54 ± 0.03). Hence, for the Monte Carlo simulation, we applied a random error of 2%. To convert DW to CW, we found several reported values for zooplankton: Banse (1996) suggested 0.4 based on two studies, Dam and Peterson (1993) assumed 0.4, and Andersen and Hessen (1991) and Andersen et al. (2016) measured 0.48 (range 0.40 - 0.57) based on measurements for 6 species. As most contemporary studies use the conversion factor 0.4, we also used this value as our mean but assumed a generous error (20%); hence we randomly sampled from a distribution of 0.40 ± 0.08.

Micronekton carbon weight (CW_micronekton_) was estimated by converting the total WW (∑WW) (Eq. 3):

CW_micronekton_ = ∑WW ± δWW ⨉ CF_DW_ ± δ_DW_ ⨉ CF_CW_ ± δ_CW_ (Eq. 3)

The precision of the balance was 2 g, which we included in the Monte Carlo simulation (δWW = 2 g). For the conversion factor for WW to DW (CF_DW_), we only found one study that provides a direct conversion of WW to DW for decapods (Pakhomov et al., 2019). These authors did not provide an uncertainty estimate for this conversion factor; however, the regression line was highly significant with an R^2^ of 0.976. We hence assume a relatively low uncertainty (δ_DW_) of 5%, and thus randomly sampled from a mean of 0.18 with a standard deviation of 0.01. For fish, López Pérez et al. (2020) provide mean water content for 36 fish species from the subtropical Atlantic (0.77 ± 0.04; see their Table 3). We hence assumed a CF_DW_ of 0.23 ± 0.04 and randomly sampled from this distribution for our Monte Carlo simulation. For the conversion factor for DW to CW (CF_CW_), we could not find any observations for micronekton, so we used the same distribution as explained above. Conversion factors used in this study are summarized in Supp. Table 2.

All CW estimates need to be corrected for potential net avoidance, which is commonly referred to as ‘capture efficiency’ (CE). A capture efficiency of 100% implies that all organisms in the water column were caught in the net. CE of 100% is commonly assumed for zooplankton collected, as the case here, with MOCNESS (Skjoldal et al., 2013). For the net we used for micronekton (Mesopelagos), there are - to our knowledge - no published data on CE. Reported values for CE of midwater trawls are 6-13% (Gjøsaeter, 1984; May and Blaber, 1989), 14% (Koslow et al., 1997), 14-38% (Davison, 2011), and 33% (Pakhomov et al., 2019). Hence, we applied a mean of 20 ± 13% in the Monte Carlo simulation.

For the sensitivity analysis of zooplankton and micronekton metabolic rate estimates, we employed the Monte Carlo method, integrating the conversion factors used for active flux conversions. Zooplankton ETS activity obtained as protein content was converted to DW using the ratio of 2.49 ± 1.73 given by Hernández-León et al. (2019c), hence assuming an error of 69%, and DW was converted to carbon as explained above. Micronekton protein content was converted to DW using the ratio given by Bailey et al. (1995) of 2.21 ± 0.45 for mesopelagic fish and 2.48 ± 1.09 for decapods, and DW was converted to carbon as explained above.

ETS in carbon units was converted to respiration using the ratio of 0.5 (Hernández-León and Gómez 1996). The respiration to ETS (R/ETS) ratio in marine zooplankton reflects the scope of metabolic activity and typically ranges between 0.5 and 1 mainly depending on factors such as species, food availability and temperature (Hernández-León and Gómez, 1996; Hernández-León et al., 2019c; Couret et al., 2024). Hernández-León et al. (2019c) observed a R/ETS value of 0.96 ± 0.29 for migrant copepods and Couret et al. (2024) obtained a ratio of 0.81 ± 0.18 for euphausiids. To maintain a conservative approach, and generally all studies adopt a conversion factor of 0.5, we also used this value as our mean but assumed a generous error (20%); hence we randomly sampled from a distribution of 0.50 ± 0.10. Respiration was converted into carbon units using a respiratory quotient (CO_2_ respired/O_2_ consumed) of 0.97 (Omori and Ikeda, 1984). The respiratory quotient is a stoichiometric consequence of the elemental composition of the substrate being oxidized. These values span the range of 0.67 to 1.24 (0.955 ± 0.4), being the calculated value for protein is 0.97 (Hernández-León and Ikeda, 2005). Hence, for the Monte Carlo simulation we assumed a respiratory quotient ratio 0.97 ± 0.4. Respiration in carbon units at depth to respiration flux is estimated following Eq. S3, that is by multiplying respiration by the migrant biomass, assuming a mesopelagic residence time of 12 h (Ariza et al., 2015).

Mortality flux was estimated from the respiration flux using a factor of 0.75 (Omori and Ikeda, 1984) for zooplankton and 0.66 (Brett and Groves, 1979) for micronekton. Estimation of zooplankton mortality rates in field populations is a challenging task that depends on multiple factors such as the swimming behavior, and consequently in vulnerability to predators (Ohman, 2012), the developmental stage composition, and the environmental variables (Ohman, 2012). Thus, we assumed a conservative error of 25%; thus, values were sampled randomly from a distribution of 0.75 ± 0.19 and 0.66 ± 0.17, respectively.

Gut flux was estimated assuming that feeding is 2.5 times respiration (Ikeda and Motoda, 1978), and that migrant zooplankton egested 50% of the gut content at depth (Ariza et al., 2015). Feeding equation depends on digestion efficiency and gross growth efficiency which can differ to a great degree, not only among zooplankton species but also within a single species (see Table 3 of Ikeda and Motoda, 1978). The latter authors chose values of 70% for digestion and 30% for gross growth as realistic values of zooplankton in the field, regardless of species and food habit. As this is the general equation used broadly to estimate gut flux from respiration, we used the value of 2.5 with a cautious assumption of a 20% error; thus, values were sampled randomly from a distribution of 2.5 ± 0.5. We assumed that migrant zooplankton egest 50% at depth following Ariza et al. (2015) estimations, as gut clearance only takes a few minutes in copepods (Dam and Peterson, 1988) and about 30-90 min in euphausiids (Gurney et al., 2002, Pakhomov et al., 2004), while in fish, estimates range from 12 h to days (Baird et al., 1975). Following Ariza et al. (2015) estimations, and assuming a nocturnal distribution at roughly a 50 m depth and a mean downwards migration velocity of 5 cm·s^−1^ (Davison et al., 2013), organisms trespassing the base of the mixed layer (∼150 m depth) should take about 30 min. This suggests that copepods will actively export relatively low amounts of fecal matter, while active swimmers like euphausiid gut flux will be partial. In any case, to be conservative, we assumed that zooplankton egested 50% at depth. Hence, for the Monte Carlo simulation, we applied a random error of 20% (assuming a normal distribution with a mean of 0.5 and a standard deviation of 0.1). For micronekton, gut flux was estimated assuming an egestion equivalent to 80% of the respired carbon (Ariza et al., 2015). Thus, we assumed a normal distribution with a mean of 0.8 and a standard deviation of 0.16 (error of 20%).

Finally, excretion flux was estimated using the values by Steinberg et al. (2000), who reported that excretion makes up 24% of the respired plus excreted carbon. On average, excretion of dissolved organic carbon makes up 24% (range=5-42%) of the total carbon metabolized (excreted + respired) (Steinberg et al., 2000). Thus, we assumed a normal distribution of 24 ± 26%.

**References**

Andersen, K.H., Berge, T., Gonçalves, R.J., Hartvig, M., Heuschele, J., Hylander, S., … Kiørboe, T. (2016). Characteristic sizes of life in the oceans, from bacteria to whales. *Annu. Rev. Mar. Sci.*, 8(1), 217–241. https://doi.org/10.1146/annurev-marine-122414-034144

Andersen, T., and Hessen, D.O. (1991). Carbon, nitrogen, and phosphorus content of freshwater zooplankton. *Limnol. Oceanogr.*, 36, 807–814.

Ariza, A., Garijo, J.C., Landeira, J.M., Bordes, F., and Hernández-León, S. (2015). Migrant biomass and respiratory carbon flux by zooplankton and micronekton in the subtropical northeast Atlantic Ocean (Canary Islands). *Prog. Oceanogr.*, 134, 330–342. https://doi.org/10.1016/j.pocean.2015.03.003

Bailey, T.G., Youngbluth, M.J., and Owen, G.P. (1995). Chemical composition and metabolic rates of gelatinous zooplankton from midwater and benthic boundary layer environments off Cape Hatteras, North Carolina, USA. *Mar. Ecol. Prog. Ser.*, 122, 121–134. https://doi.org/10.3354/meps122121

Baird, R., Thompson, N., Hopkins, T., and Weiss, W. (1975). Chlorinated hydrocarbons in mesopelagic fishes of the eastern Gulf of Mexico. *Bull. Mar. Sci.*, 25, 473–481.

Banse, K. (1996). Zooplankton: pivotal role in the control of ocean production. *Oceanogr. Lit. Rev.*, 5, 455.

Bárcena, M., Flores, J., Sierro, F., Pérez-Folgado, M., Fabres, J., Calafat, A., and Canals, M. (2004). Planktonic response to main oceanographic changes in the Alboran Sea (Western Mediterranean) as documented in sediment traps and surface sediments. *Mar. Micropaleontol.*, 53, 423–445.

Brett, J.R., and Groves, T.D.D. (1979). Physiological energetics. In: Fish Physiology (Vol. 8, pp. 280–352).

Couret, M., Díaz-Pérez, J., Sarmiento-Lezcano, A.N., Landeira, J.M., and Hernández-León, S. (2024). Respiration rates and its relationship with ETS activity in euphausiids: implications for active flux estimations. *Front. Mar. Sci.*, 11. https://doi.org/10.3389/fmars.2024.1469587

Dam, H.G., and Peterson, W.T. (1988). The effect of temperature on the gut clearance rate constant of planktonic copepods. *J. Exp. Mar. Biol. Ecol.*, 123, 1–14. https://doi.org/10.1016/0022-0981(88)90105-0

Dam, H.G., and Peterson, W.T. (1993). Seasonal contrasts in the diel vertical distribution, feeding behavior, and grazing impact of the copepod Temora longicornis in Long Island Sound. *J. Mar. Res.*, 51, 561–594. https://doi.org/10.1357/0022240933223972

Davison, P. (2011). The specific gravity of mesopelagic fish from the Northeastern Pacific Ocean and its implications for acoustic backscatter. *ICES J. Mar. Sci*. https://doi.org/10.1093/icesjms/fsr140

Davison, P.C., Checkley, D.M., Koslow, J.A., and Barlow, J. (2013). Carbon export mediated by mesopelagic fishes in the northeast Pacific Ocean. *Prog. Oceanogr.*, 116, 14–30. https://doi.org/10.1016/j.pocean.2013.05.013

Fiuza, A.F. (1983). Upwelling patterns off Portugal. In: Suess, E., and Thiede, J. (Eds.) Coastal Upwelling: Its Sediment Record, Part A (pp. 85–98). Springer US. https://doi.org/10.1007/978-1-4615-6651-9_5

Gjøsaeter, J. (1984). Mesopelagic fish, a large potential resource in the Arabian Sea. *Deep Sea Res.*, 31, 1019–1035. https://doi.org/10.1016/0198-0149(84)90054-2

Gurney, L., Froneman, P., Pakhomov, E., and McQuaid, C. (2002). Diel feeding patterns and daily ration estimates of three Subantarctic euphausiids in the vicinity of the Prince Edward Islands (Southern Ocean). *Deep-Sea Res. II*, 49, 3207–3227.

Hays, G.C. (2003). A review of adaptive significance and ecosystem consequences of zooplankton diel vertical migrations. *Hydrobiologia*, 503, 163–170.

Hernández-León, S., and Gómez, M. (1996). Factors affecting the respiration/ETS ratio in marine zooplankton. J*. Plankton Res.*, 18, 239–255. https://doi.org/10.1093/plankt/18.2.239

Hernández-León, S., and Ikeda, T. (2005). Zooplankton respiration. In: del Giorgio, P.A., and Williams, P.J.l.B. (Eds.) Respiration in aquatic ecosystems (pp. 67–82). Oxford: Oxford Univ. Press.

Hernández-León, S., Calles, S., and Fernández de Puelles, M.L. (2019). The estimation of metabolism in the mesopelagic zone: Disentangling deep-sea zooplankton respiration. *Prog. Oceanogr.*, 178, 102163. https://doi.org/10.1016/j.pocean.2019.102163

Ikeda, T. (1989). Estimated respiration rate of myctophid fish from the enzyme activity of the electron-transport-system. *J. Oceanogr. Soc. Jpn.,* 45, 167–173.

Ikeda, T., and Motoda, S. (1978). Estimated zooplankton production and their ammonia excretion in the Kuroshio and adjacent seas. *Fish. Bull.*, 76, 357–367.

King, F.D., and Packard, T.T. (1975). Respiration and the activity of the respiratory electron transport system in marine zooplankton. *Limnol. Oceanogr.*, 20, 849–854. https://doi.org/10.4319/lo.1975.20.5.0849

Koslow, J.A., Kloser, R.J., and Williams, A. (1997). Pelagic biomass and community structure over the mid-continental slope off southeastern Australia based upon acoustic and mid-water trawl sampling. *Mar. Ecol. Prog. Ser.*, 146, 21–35.

Lehette, P., and Hernández-León, S. (2009). Zooplankton biomass estimation from digitized images: a comparison between subtropical and Antarctic organisms. *Limnol. Oceanogr. Methods*, 7, 304–308. https://doi.org/10.4319/lom.2009.7.304

López-Pérez, C., Olivar, M.P., Hulley, P.A., and Tuset, V.M. (2020). Length–weight relationships of mesopelagic fishes from the equatorial and tropical Atlantic waters: influence of environment and body shape. *J. Fish Biol.*, 96, 1388–1398. https://doi.org/10.1111/jfb.14307

Lowry, O.H., Rosebrough, N.J., Farr, A.L., and Randall, R.J. (1951). Protein measurement with the Folin phenol reagent. *J. Biol. Chem.*, 193, 265–275.

Massutí, E., Sánchez-Guillamón, O., Farriols, M.T., Palomino, D., Frank, A., Bárcenas, P., … López-Rodríguez, C. (2021). Improving scientific knowledge of Mallorca Channel seamounts (Western Mediterranean) within the framework of Natura 2000 network. *Diversity*, 14(1), 4.

May, J.L., and Blaber, S.J.M. (1989). Benthic and pelagic fish biomass of the upper continental slope off eastern Tasmania. *Mar. Biol.*, 101, 11–25.

Ohman, M.D. (2012). Estimation of mortality for stage-structured zooplankton populations: What is to be done? *J. Mar. Syst.*, 93, 4–10.

Omori, M., and Ikeda, T. (1984). Methods in Marine Zooplankton Ecology. New York, NY: John Wiley and Sons.

Pakhomov, E.A., and Froneman, P.W. (2004). Zooplankton dynamics in the eastern Atlantic sector of the Southern Ocean during the austral summer 1997/1998—Part 1: Community structure. *Deep-Sea Res. II*, 51, 2599–2616.

Penaud, A., Eynaud, F., Voelker, A.H.L., and Turon, J.L. (2016). Palaeohydrological changes over the last 50 ky in the central Gulf of Cadiz: Complex forcing mechanisms mixing multi-scale processes. *Biogeosciences*, 13, 5357–5377. https://doi.org/10.5194/bg-13-5357-2016

Rutter, W.J. (1967). Methods in developmental biology. In: Wilt, H.F., and Wessels, N.K. (Eds.) Methods in Developmental Biology (pp. 671–684). New York: Academic Press.

Skjoldal, H.R., Wiebe, P.H., Postel, L., Knutsen, T., Kaartvedt, S., and Sameoto, D.D. (2013). Intercomparison of zooplankton (net) sampling systems: Results from the ICES/GLOBEC sea-going workshop. *Prog. Oceanogr*., 108, 1–42. https://doi.org/10.1016/j.pocean.2012.10.006

Steinberg, D.K., Carlson, C.A., Bates, N.R., Goldthwait, S.A., Madin, L.P., and Michaels, A.F. (2000). Zooplankton vertical migration and the active transport of dissolved organic and inorganic carbon in the Sargasso Sea. *Deep Sea Res. I,* 47, 137–158. https://doi.org/10.1016/S0967-0637(99)00052-7

Villegas-Ríos, D., Álvarez-Salgado, X.A., Piedracoba, S., Rosón, G., Labarta, U., and Fernández-Reiriz, M.J. (2011). Net ecosystem metabolism of a coastal embayment fertilised by upwelling and continental runoff. *Cont. Shelf Res*., 31, 400–413.
