# Supplementary material for: Integration of bulk and single-cell transcriptomic data reveals a novel signature related to liver metastasis and basement membrane in pancreatic cancer
Source: Front Immunol. 2025 Oct 29;16:1671956. doi: 10.3389/fimmu.2025.1671956 (PMC12605406; doi:10.3389/fimmu.2025.1671956)
Supplement: Supplementary file 13 [file Table1.docx]

| **Table S1**. The information of datasets in this study | | | |
| --- | --- | --- | --- |
| Database | Dataset | Description | Characteristics |
| TCGA | PAAD | 178 tumor samples, 4 non-tumor samples | bulk transcriptome |
| GTEx | Pancreas | 167 non-tumor samples | bulk transcriptome |
| ICGC | PA_AU | 255 tumor samples | bulk transcriptome |
| GEO | GSE71729 | 145 primary PDAC samples and 25 liver metastasis samples | bulk transcriptome |
|  | GSE34153 | 14 primary tumor samples and 20 liver metastatic samples | bulk transcriptome |
|  | GSE197177 | 3 primary pancreatic tumor samples, 3 liver metastatic samples and 1 normal pancreatic tissue | single cell transcriptome |
|  | GSE154778 | 10 primary pancreatic tumor samples and 6 liver metastatic samples | single cell transcriptome |
