# Supplementary material for: Integration of bulk and single-cell transcriptomic data reveals a novel signature related to liver metastasis and basement membrane in pancreatic cancer
Source: Front Immunol. 2025 Oct 29;16:1671956. doi: 10.3389/fimmu.2025.1671956 (PMC12605406; doi:10.3389/fimmu.2025.1671956)
Supplement: Supplementary file 14 [file Table2.docx]

| **Table S2**. Specific parameters of each method for predicting the prognosis | | |
| --- | --- | --- |
| **Method** | **Parameters** | **Value** |
| RSF | ntree | 1000 |
|  | nodesize | 5 |
|  | splitrule | logrank |
|  | importance | TRUE |
|  | proximity | TRUE |
|  | forest | TRUE |
| Enet | alpha | 0.1, 0.2, 0.3, 0.4, 0.5, 0.6, 0.7, 0.8, 0.9 |
|  | family | cox |
|  | nfolds | 10 |
| StepCox | direction | both, backward, forward |
| CoxBoost | trace | TRUE |
|  | start.penalty | 500 |
|  | maxstepno | 500 |
|  | K | 10 |
|  | type | verweij |
| plsRcox | nt | 10 |
| SuperPC | n.fold | 10 |
|  | n.components | 3 |
|  | max.features | nrow(data) |
|  | compute.fullcv | TRUE |
|  | compute.preval | TRUE |
|  | min.features | 2 |
| GBMs | distribution | coxph |
|  | n.trees | 10000 |
|  | interaction.depth | 3 |
|  | n.minobsinnode | 10 |
|  | shrinkage | 0.001 |
|  | cv.folds | 10 |
| SurvivalSVM | gamma.mu | 1 |
| Ridge | alpha | 0 |
|  | nfold | 10 |
|  | family | cox |
| Lasso | alpha | 1 |
|  | nfold | 10 |
|  | family | cox |
| Other parameters were set as default values | | |
